# Human cochlear microstructures at risk of electrode insertion trauma, elucidated in 3D with contrast-enhanced microCT

**DOI:** 10.1038/s41598-023-29401-6

**Published:** 2023-02-07

**Authors:** Anastasiya Starovoyt, Grzegorz Pyka, Tristan Putzeys, Tim Balcaen, Jan Wouters, Greet Kerckhofs, Nicolas Verhaert

**Affiliations:** 1 ExpORL, Department of Neurosciences, Katholieke Universiteit (KU) Leuven, 3000 Leuven, Belgium; 2 Department of Neurosciences, Leuven Brain Institute, KU Leuven, 3000 Leuven, Belgium; 3 Biomechanics Laboratory, Institute of Mechanics, Materials, and Civil Engineering, Université Catholique de (UC) Louvain, 1348 Louvain-la-Neuve, Belgium; 4 Laboratory for Soft Matter and Biophysics, Department of Physics and Astronomy, KU Leuven, 3000 Leuven, Belgium; 5 Molecular Design & Synthesis, Department of Chemistry, KU Leuven, 3001 Leuven, Belgium; 6 Prometheus, Division of Skeletal Tissue Engineering, KU Leuven, 3000 Leuven, Belgium; 7 Department of Materials Science and Engineering, KU Leuven, 3000 Leuven, Belgium; 8 Institute of Experimental and Clinical Research, UC Louvain, 1200 Woluwé-Saint-Lambert, Belgium; 9 Department Otorhinolaryngology, Head and Neck Surgery, University Hospitals of Leuven, 3000 Leuven, Belgium; 10 Research Group Experimental Oto-Rhino-Laryngology, O&N II Herestraat 49, Bus 721, 3000 Leuven, Belgium

**Keywords:** Cochlea, Anatomy, Translational research

## Abstract

Cochlear implant restores hearing loss through electrical stimulation of the hearing nerve from within the cochlea. Unfortunately, surgical implantation of this neuroprosthesis often traumatizes delicate intracochlear structures, resulting in loss of residual hearing and compromising hearing in noisy environments and appreciation of music. To avoid cochlear trauma, insertion techniques and devices have to be adjusted to the cochlear microanatomy. However, existing techniques were unable to achieve a representative visualization of the human cochlea: classical histology damages the tissues and lacks 3D perspective; standard microCT fails to resolve the cochlear soft tissues; and previously used X-ray contrast-enhancing staining agents are destructive. In this study, we overcame these limitations by performing contrast-enhanced microCT imaging (CECT) with a novel polyoxometalate staining agent Hf-WD POM. With Hf-WD POM-based CECT, we achieved nondestructive, high-resolution, simultaneous, 3D visualization of the mineralized and soft microstructures in fresh-frozen human cochleae. This enabled quantitative analysis of the true intracochlear dimensions and led to anatomical discoveries, concerning surgically-relevant microstructures: the round window membrane, the Rosenthal’s canal and the secondary spiral lamina. Furthermore, we demonstrated that Hf-WD POM-based CECT enables quantitative assessment of these structures as well as their trauma.

## Introduction

Over 5% of the world’s population and over 25% of individuals older than 60 years suffer from disabling hearing loss^1^. In most cases, hearing loss is caused by abnormal function of the cochlea. Cochlear dysfunction results in impaired understanding of speech, which is highly invalidating for professional, social, emotional and cognitive well-being, and cannot be sufficiently rehabilitated with hearing aids^2^. Cochlear implants (CIs) can restore speech understanding by converting sounds into electrical pulses and directly stimulating the auditory nerve within the cochlea^3^. However, the stimulating electrode array of the CI has to be surgically inserted into the cochlea. Electrode insertion can damage intracochlear structures and compromise the remaining hearing, which is important for hearing in a noisy environment and for the appreciation of music^4^. According to a recent systematic review, electrode insertion trauma occurs in up to 32% of implantations^5^. To avoid electrode insertion trauma, surgical techniques and electrode array design have to be perfectly tailored to the cochlear microanatomy^6–8^.

Different structures can be traumatized during electrode insertion. While on the outside, the small—typically around 4 mm × 7 mm × 10 mm^9^—cochlea is protected by a thick bony capsule, on the inside it consists of very delicate structures. The internal cochlear lumen is filled with fluid and rolled up in a spiral with on average 2.6 turns, each turn comprising three compartments: scala tympani (ST), scala vestibuli and scala media^6,10,11^. The electrode is typically inserted into the ST through a soft-tissue round window membrane (RWM): the only part of the cochlea which is not covered by bone^12,13^. The ST is separated from the other compartments by three thin walls: a bony osseous spiral lamina (OSL), a soft-tissue cochlear partition bridge (CPB) and a soft basilar membrane (BM)^11,14^. If the insertion trajectory deviates from the 3D shape of the ST, these walls can be traumatized and, in some cases, the electrode can transgress into the other two compartments. In addition, the outer and the inner wall of the ST can be at risk of trauma^15–20^. The outer wall is occupied by the spiral ligament (SL), which is responsible for the blood supply to the cochlea. The bony central cone of the cochlea, called the modiolus, is located behind the inner wall and contains hearing neurons, innervating the cochlea from within the Rosenthal’s canal (RC), which is positioned adjacent to the ST. The fibers of these neurons run from the hearing organ of Corti (OoC) through the OSL to the RC. The OoC contains the hearing receptor cells and sits mostly on top of the CPB and the BM inside the scala media^14^. Damage to the hearing receptor cells of the OoC, the capillary network of the SL, the neural fibers inside the OSL or the neurons inside the RC, as well as perforations of the scala media compartment, can result in immediate loss of residual hearing after CI surgery. Electrode transgression outside the ST compartment is always accompanied by perforation of at least one of the cochlear walls separating it from the other compartments (OSL, CPB, BM or SL). Additionally, transgression from the ST results in a larger distance between the electrode array and the hearing neurons within the RC, leading to suboptimal performance of the CI. Any trauma can also trigger chronic inflammation, followed by fibrosis and ossification inside the cochlear compartments, which often leads to delayed deterioration of the remaining hearing and suboptimal stimulation by the electrode array^4,18^.

Understanding the precise 3D anatomy of the ST and its delicate walls is crucial to avert electrode insertion trauma. Yet, anatomical study of the human cadaveric cochlea is challenging, due to the bony capsule surrounding it and prohibiting direct visualization of the intracochlear space. Our current insights into the cochlear microanatomy are predominantly based on classical histological analysis of fixed human cadaveric specimens^11,21–27^, whereby the cochlea is physically cut into thin slices, which are then (immuno-) stained and viewed under a microscope. Classical histology enables clear, high-resolution visualization of the intracochlear structures, but it is limited by the cutting angle and the thickness of the 2D sections. Furthermore, histological preparation requires extensive tissue processing, which results in shrinkage, deformations and oftentimes damage to the delicate cochlear membranes^21–25^. Therefore, visualization obtained with classical histology is not entirely representative for the 3D anatomy of a fresh human cochlea.

Intracochlear imaging with microfocus X-ray computed tomography (microCT) overcomes the shortcomings of classical histological analysis by enabling nondestructive 3D visualization of the entire cochlea^6,9,28–30^. However, X-ray-based imaging modalities are restricted by the limited contrast in soft tissues. Due to this limitation, intracochlear soft-tissue structures cannot be discerned from the surrounding fluid on standard microCT images. To tackle this, intracochlear fluid can be aspirated through the RWM before imaging^6,28,31^. Although, full removal of the fluid is not always possible and soft tissues can be easily deformed in the process of aspiration: the geometry of the RWM alters when punctured and the scala media compartment, which is fully delineated by soft-tissue BM and the Reissner’s membrane, often collapses and cannot be discerned from the SL. A viable alternative is contrast-enhanced microCT (CECT), which makes use of contrast-enhancing staining agents (CESAs) to increase the soft tissue X-ray attenuation. Yet, the common limitation of all previously used CESAs for intracochlear imaging (osmium tetroxide, Lugol’s iodine and phosphotungstic acid) is that they were inherently destructive for the tissue^23,32–35^. Furthermore, so far, CECT was only performed on fixed samples, whereby soft tissue structures were already affected by shrinkage^24,25^.

The objective of this study was to obtain simultaneous 3D visualization of the mineralized and soft-tissue intracochlear structures, which are relevant for CI surgery, in fresh human cadaveric cochleae in a fully nondestructive manner. To achieve this, we made use of a promising CESA, which has been recently introduced for soft tissue visualization on CECT: a 1:2 hafnium-substituted Wells–Dawson polyoxometalate (K_16_[Hf(α_2_-P_2_W_17_O_61_)_2_]·19H_2_O), further referred to as Hf-WD POM^36–39^. Similar to phosphotungstic acid, Hf-WD POM has a binding affinity for fibrin, collagen and fibers of connective tissue. However, thanks to its near-physiological pH, Hf-WD POM solution is not destructive for the tissue; this was previously validated in fixed murine samples^36^. Here, we took the next step and applied Hf-WD POM-based CECT on fresh-frozen human cochleae, which are considered most representative of a living cochlea for preclinical electrode insertion studies^40,41^. For the purpose of CI surgery, we were primarily interested in visualizing the intracochlear structures at risk of electrode insertion trauma (OSL, CPB, BM, SL and RC) and the 3D anatomy of the RWM, which is the main surgical entry point to the cochlea. In addition, special attention was given to the anatomy of the secondary spiral lamina (SSL), a bony protrusion in the outer cochlear wall, which accompanies the SL in the proximal part of the ST. Agrawal et al. recently reported that the anatomy of the SSL should be considered for atraumatic electrode insertion in cochlear implantation^42^, but this structure had not yet been intensively studied in the human cochlea. Finally, we also investigated whether Hf-WD POM-based CECT enables 3D visualization and quantitative analysis of electrode insertion trauma in human cadaveric cochleae.

## Results

### Soft cochlear membranes visualized alongside mineralized bone with Hf-WD POM-based CECT

To achieve a realistic visualization of the cochlear microanatomy, its mineralized and soft-tissue structures have to be imaged in 3D without any alterations by either mechanical manipulation or (bio)chemical degradation. Standard microCT can image the mineralized structures of the cochlea (cochlear capsule, modiolus, RC, OSL and SSL), but not the soft-tissue membranes (RWM, BM, SL, CPB-OoC, RM). We hypothesized that Hf-WD POM would be able to bind to the cochlear soft-tissue structures, which contain collagen amongst other connective tissue fibers^11,43^, resulting in simultaneous visualization of soft and mineralized intracochlear structures on CECT images. We assumed that the human cochleae can be stained nondestructively with Hf-WD POM by submersion, whereby the CESA would diffuse into the intracochlear fluid through the RWM^44^.

**Figure 1:**
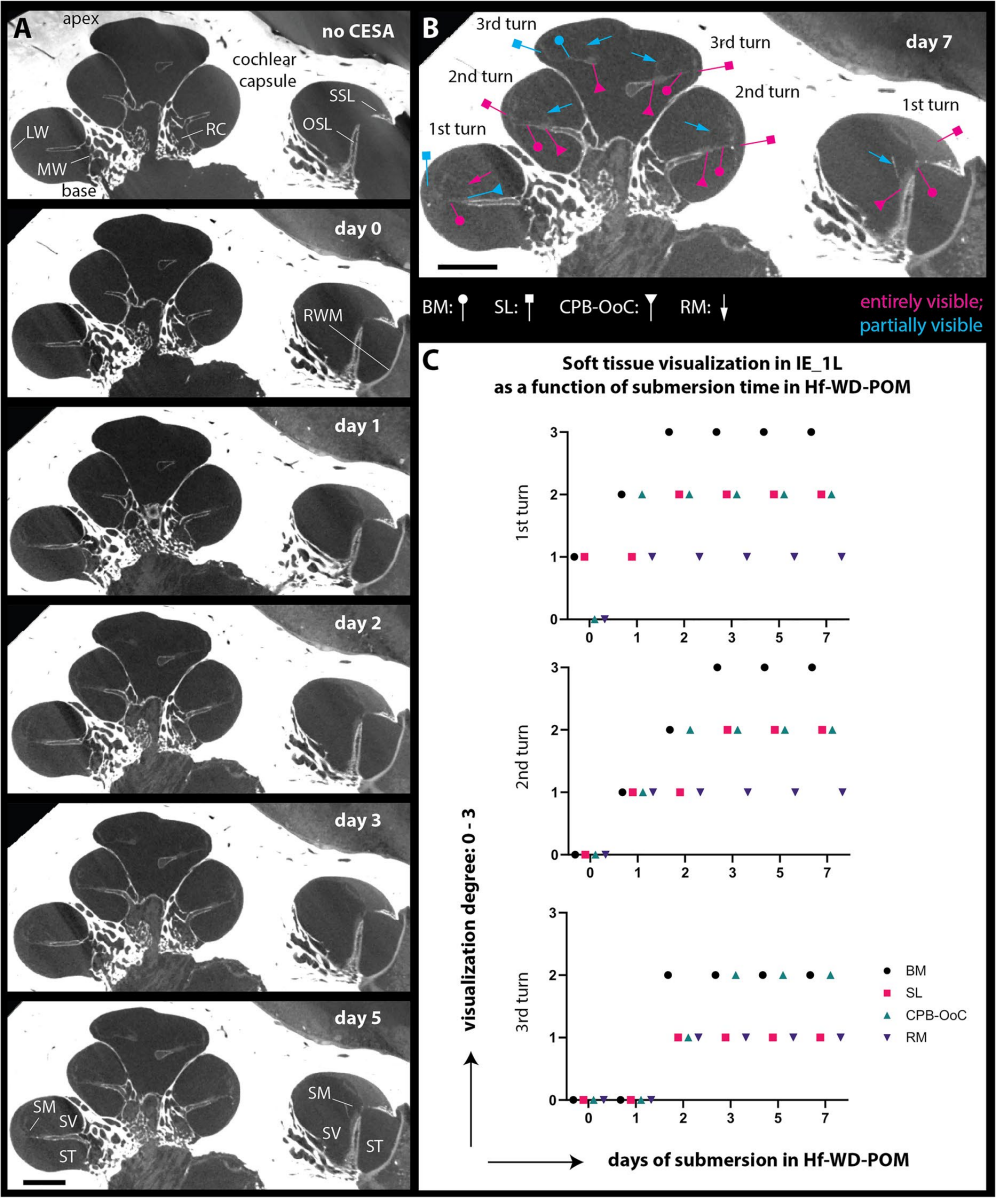
Hf-WD POM-related soft tissue visualization in IE_1L. (**A**) Visualization of the bony and soft intracochlear structures on transmodiolar cross-sections during CESA follow-up study in IE_1L. Without CESA, only mineralized structures could be visualized; increasing contrast of the soft tissues was achieved after submersion in Hf-WD POM solution (day 0–5). Day 0 dataset was acquired within three hours after submersion. (**B**) Transmodiolar cross-section on day 7 illustrates assessment of soft tissue visualization. (**C**) Graphical representation of soft tissue visualization in each cochlear turn, as a function of submersion time in Hf-WD POM. By assessing all slices of each dataset, as demonstrated in (**B**), soft tissue visualization was graded as follows: 0, not visible; 1, partially visible on some slices; 2, partially visible on all slices; 3: entirely visible on all slices. BM: basilar membrane; CPB-OoC: cochlear partition bridge—organ of Corti complex; LW: lateral wall; MW: modiolar wall; OSL: osseous spiral lamina; RC: Rosenthal’s canal; RM: Reissner’s membrane; RWM: round window membrane; SL: spiral ligament; SM: scala media; SSL: secondary spiral lamina; ST: scala tympani; SV: scala vestibuli. Scale bar: 1 mm.

The experiments conducted on four fresh-frozen human ears (Supplementary Fig. 1) confirmed these hypotheses: the RWM was stained within three hours after submersion in Hf-WD POM, and the other intracochlear soft tissues progressively gained contrast from base to apex, approximately one cochlear turn per day. Full staining of all cochlear turns was achieved after 3 days of submersion, whereby the structures of interest appeared unaffected by biochemical decay (Fig. 1, Supplementary Fig. 2).

### Hf-WD POM-based CECT imaging validated by comparison with histology and literature

To make sure that Hf-WD POM-based CECT is a reliable method for imaging of the intracochlear structures, we compared the visualized structures on CECT images to classical histology (qualitative validation) and the measured dimensions of the cochleae to the literature (quantitative validation).

Qualitative comparison of the histological slices to their respective CECT images revealed clear visualization of the same intracochlear structures (Movie 1, Supplementary Fig. 3). However, the thin bony and especially soft-tissue structures often appeared damaged or deformed on the histological sections, highlighting the need for a nondestructive imaging technique, such as CECT.

Quantitative analysis of the cochlear dimensions (Table 1), showed that these were in line with previous studies^6,9,10,21,45–56^. This demonstrates that Hf-WD POM-based CECT imaging is a reliable method for quantitative analysis of the cochlear dimensions.

**Table 1:** Overview of the cochlear dimensions in the four cochleae.

	IE_1L	IE_2L	IE_3R	IE_4R	Measured range	Range in the literature
Length (mm)	9.5	8.9	8.8	10.1	8.8–10.1	8.0–10.1^6,9,55^
Width (mm)	6.8	6.4	6.6	7.2	6.4–7.2	5.6–8.2^6,9,10,55^
Height (mm)	4.2	3.7	3.6	4.0	3.6–4.2	3.3–4.8^6,9,10^
Number of turns	2.8	2.6	2.7	2.6	2.6–2.8	2.2–2.9^6,9,10,51^
Volume (mm^3^)	81.3	73.6	75.8	102.7	73.6–102.7	50.8–118.5^51–54^

Cochlear width, length and height were measured as previously described by Shin et al.^9^. The total cochlear volume was calculated as the volume sum of the intracochlear structures (Supplementary Table 1).

### Intracochlear structures with relevance for cochlear implantation rendered and analyzed in 3D

Since the CI electrode array is typically inserted into the ST, the 3D anatomy of this compartment and its relation to the other intracochlear structures are crucial to understand the mechanics of electrode insertion trauma. Hf-WD POM-based CECT imaging enabled us to segment the separate intracochlear structures and compartments and to render them in 3D (Movie 2).

**Figure 2:**
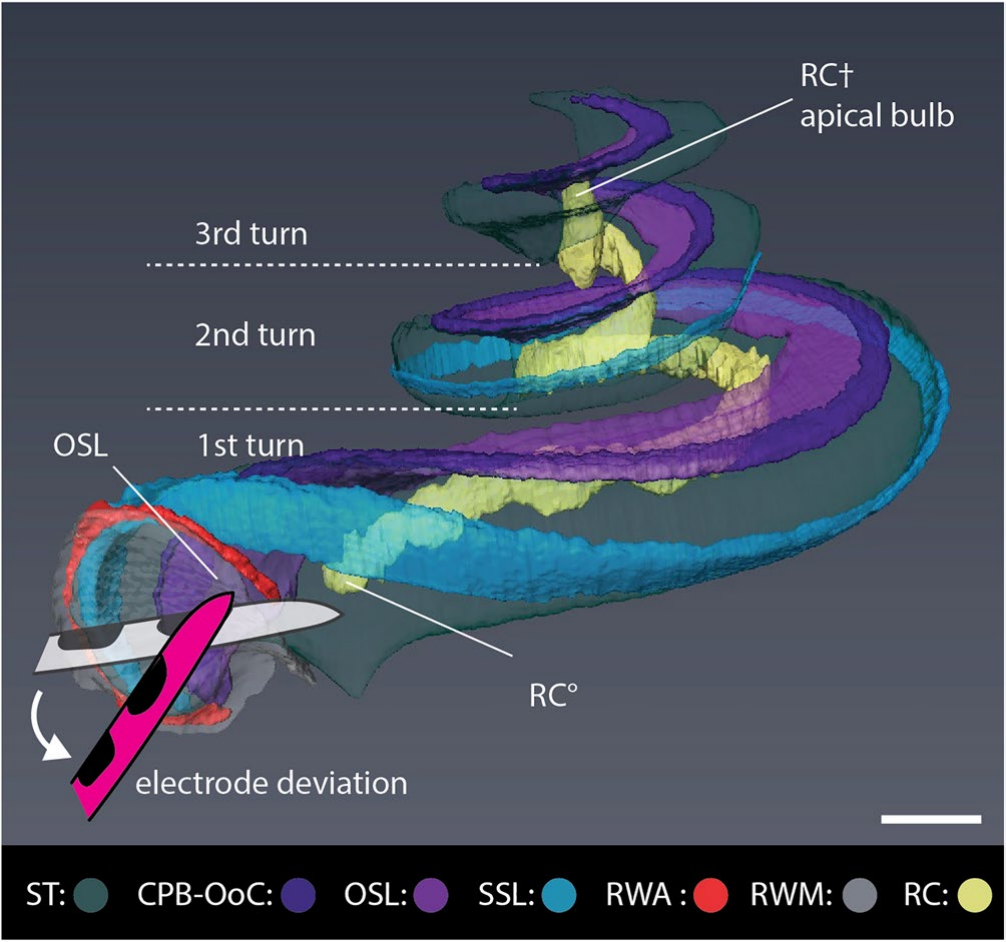
3D anatomy of proximal electrode deviation with respect to the OSL and the RC. Schematic of proximal electrode deviation superimposed on CECT-based 3D rendering of the IE_3R demonstrates that the OSL is more at risk of collision and trauma than the proximal end of the RC (RC°). Also, note the position of the distal end of the RC (RC†), a.k.a. the apical bulb, next to the ST of the 3rd cochlear turn. CPB-OoC: cochlear partition bridge—organ of Corti complex; OSL: osseous spiral lamina; RC: Rosenthal’s canal; RWA: round window arch; RWM: round window membrane; SSL: secondary spiral lamina; ST: scala tympani. Scale bar: 1 mm.

Firstly, we evaluated the 3D microanatomy of the RC, which contains the auditory neurons, stimulated by the CI. On the one hand, previous studies speculated that proximal RC may be at risk of trauma, when the electrode deviates towards the inner wall of the ST^57–59^. On the other hand, the maximal insertion depth of the electrode array is often set at the two cochlear turns, because previous studies reported that the apical end of the RC does not extend into the third turn^21,47,49,60^. On the contrary, our 3D rendering of the RC demonstrated that misalignment of the electrode insertion in the proximal cochlea is far more likely to collide with and traumatize the OSL than the RC, since the RC does not always start immediately at the level of the RWM. Additionally, 3D visualization showed that the ‘apical bulb’ was in fact consistently positioned adjacent to the ST of the third cochlear turn (Fig. 2, Supplementary Fig. 4, Movie 3). This finding implies that the RC, is present beyond the second cochlear turn, indicating that CI patients may in fact benefit from deeper electrode insertions (until the third cochlear turn), which would enable neurostimulation of the more apical low-frequency regions of the cochlea.

Secondly, we explored the possibilities of 3D quantitative analysis of the intracochlear structures, based on Hf-WD POM-based CECT. While extensive analysis was beyond the scope of our current study, we did demonstrate the feasibility of 3D measurements, some of which had not been assessed in the past: the volume of the RWM, scala media, scala vestibuli, CPB, OSL, SL, SSL, RC; the centerline length of the scala vestibuli, OSL, SL, SSL; the angular position of the proximal RC end (Supplementary Table 1). Clear visualization of the soft-tissue structures enabled us to segment the separate cochlear compartments as well as the SL with unprecedented accuracy. The measurements indicate that some intracochlear dimensions, which are relevant for CI surgery (e.g. the length of the OoC and the RC), may vary not only between but also within the individuals (the samples IE_2L and IE_3R). Additionally, several parameters were found to follow the same trend: the length and width of the cochlear base, the total cochlear volume, and the volumes of the separate compartments.

**Figure 3:**
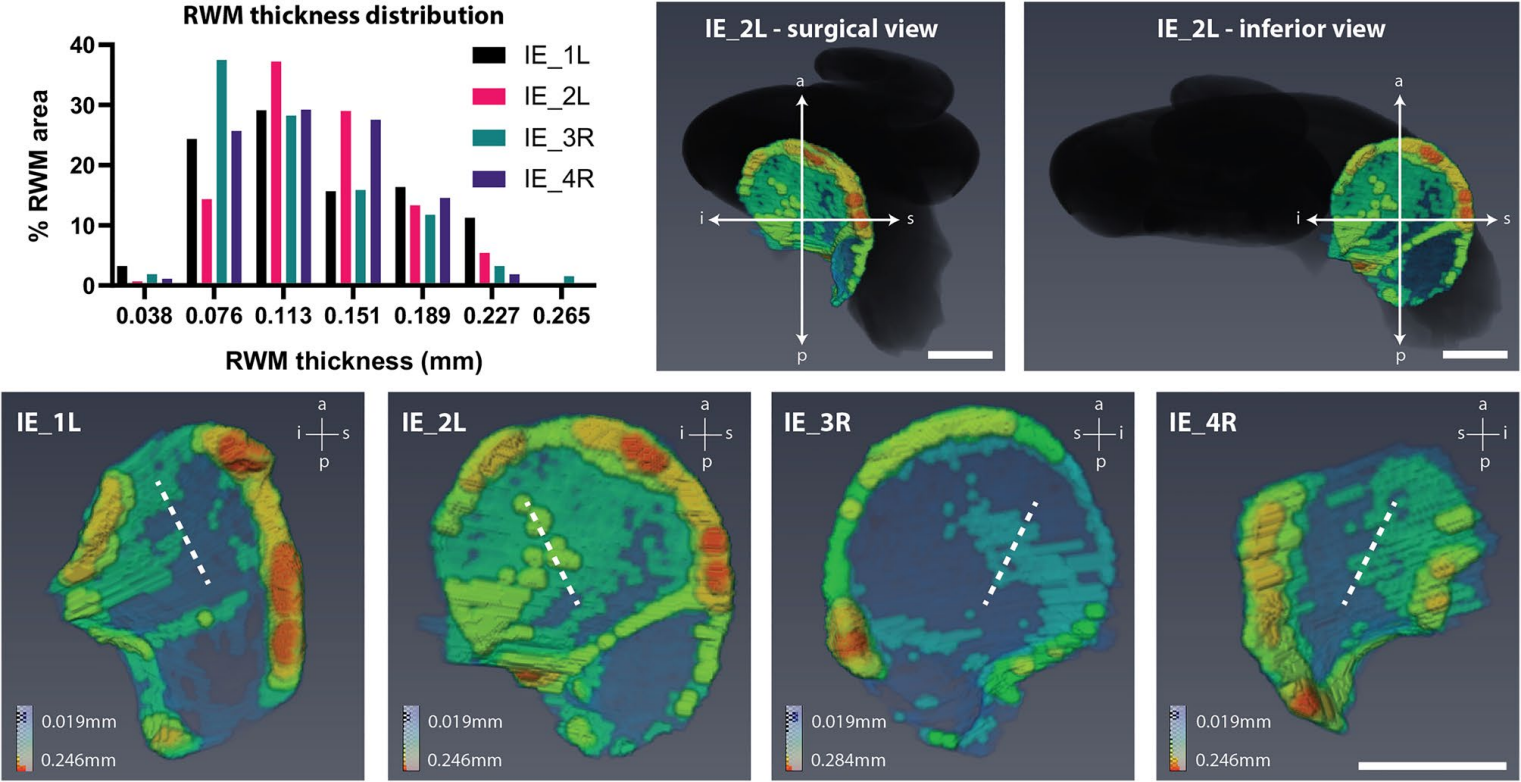
3D thickness profile of the RWM. *(top left)* Graphical presentation of the RWM thickness distribution in the four cochleae, calculated using sphere-fitting algorithm on segmented data at the resolution of 18.9 µm^3^^92^. The x-axis shows the middle value for each thickness range, e.g. 0.076 mm for the range 0.057–0.095 mm. The y-axis represents the percentage of RWM area within each thickness range. For additional information, see Supplementary Table 2. *(top center and right)* 3D rendered RWM in IE_2L with respect to the entire cochlear spiral (in black) in surgical and inferior view with indication of the anatomical orientation (a: anterior; p: posterior; i: inferior; s: superior). In the inferior view, the whole cochlea in the surgical view is rotated to the right by approx. 90°, leading to a better visualization of the RWM surface area. *(bottom)* The geometry and the thickness map of the four separate RWMs from inferior view. IE_2L and IE_3R are the left and right side in the same subject. The dashed line shows the assessed position of the surgical incision for electrode insertion through the RWM in CI surgery. RWM: round window membrane. Scale bar: 1 mm.

Finally, we studied the shape and the thickness of the RWM, since it is the most important surgical access point to the cochlea, and previous data were based on histological sections^61,62^, fixed cochleae^34^ or isolated membranes^43^, all of which already lost their natural 3D form and tension by being processed or detached from the rest of the cochlea. CECT-based segmentation of fresh-frozen RWMs for the first time allowed us to analyze the thickness profile of the entire RWM in its native shape and position. We found that the central thickness of the RWM varied between 0.057 mm and 0.17 mm in the four studied samples, with a distribution peak between 0.057 and 0.13 mm (Fig. 3, Supplementary Table 2), contrary to the previous measurements in the range of 0.06–0.07 mm^43,61,62^. The RWM thickness varied both between and within individuals, whereas the perimeter shape of the RWM was similar for the different anatomical sides in the same subject (IE_2L and IE_3R in Fig. 3).

### The true anatomy of the human secondary spiral lamina revealed… another structure

**Figure 4:**
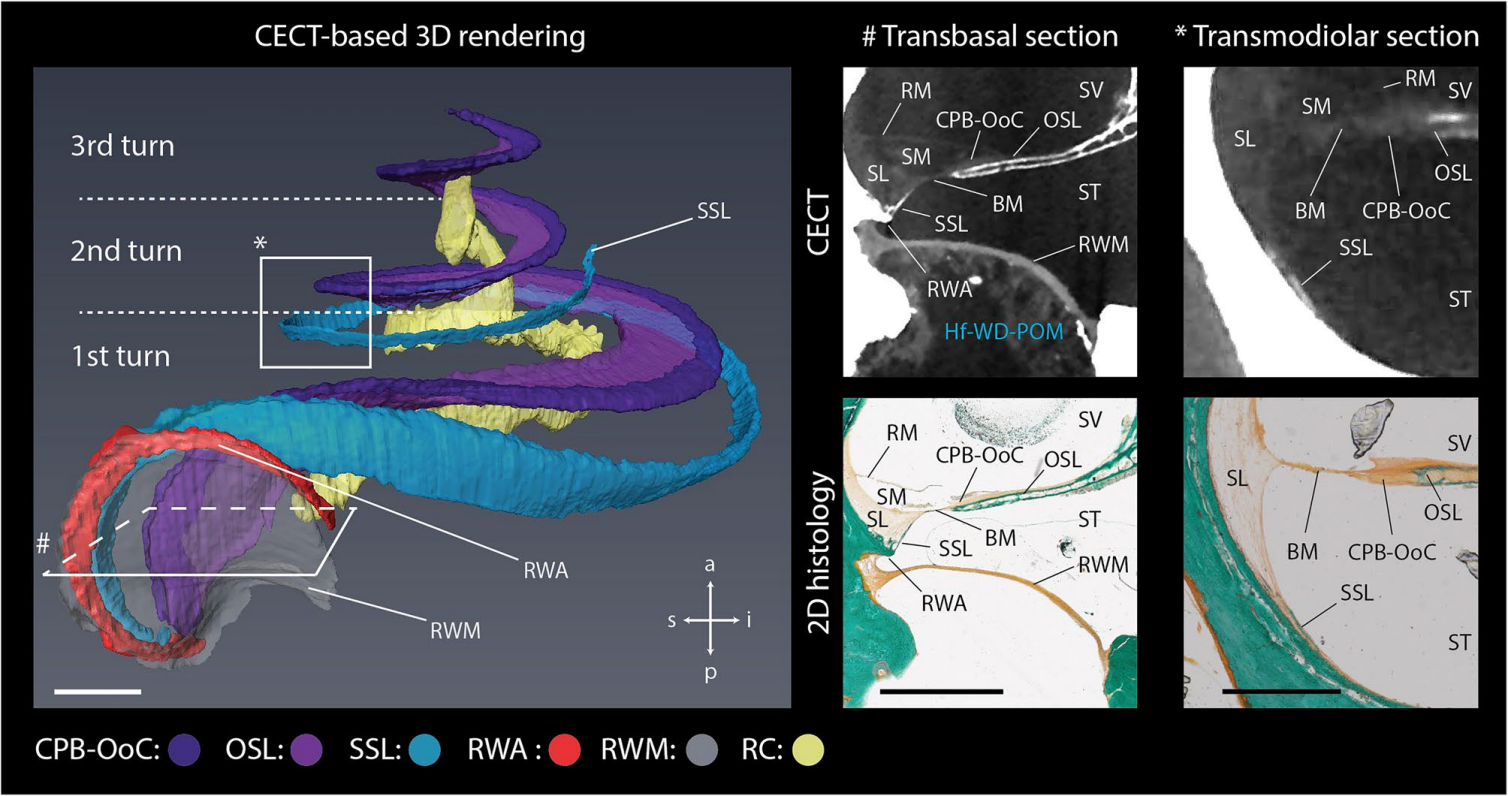
The distinct 3D anatomy of the SSL and the RWA in IE_3R. *(left)* Semi-transparent CECT-based 3D rendering of the structures in IE_3R with indication of the cochlear turns. The rectangles (#) and (*) indicate the positions of transbasal and transmodiolar sections, depicted on the right. Scale bar: 1 mm. *(right)* Transbasal and transmodiolar CECT and corresponding Masson–Goldner histological sections. The transbasal sections illustrate that the SSL is a porous microstructure, whereas the RWA, consists of dense cortical bone. The transmodiolar sections illustrate the position of the SSL at the basal attachment point of the SL approximately 270° from the center of the RWM. BM: basilar membrane; CPB-OoC: cochlear partition bridge—organ of Corti complex; OSL: osseous spiral lamina; RC: Rosenthal’s canal; RM: Reissner’s membrane; RWA: round window arch; RWM: round window membrane; SL: spiral ligament; SM: scala media; SSL: secondary spiral lamina; ST: scala tympani; SV: scala vestibuli. Scale bar 3D rendering and transbasal section: 1 mm. Scale bar transmodiolar section: 0.5 mm.

During the segmentation and the 3D rendering of intracochlear structures, special attention was given to the SSL. This structure was hypothesized to be of relevance for CI surgery, yet only a limited number studies reported on its anatomy within the human cochlea^12,42,50,59,63,64^. Contrary to these studies, which visualized the SSL exclusively adjacent to the RWM, we found that the SSL extended until the second cochlear turn (370–572°) in all four analyzed human cochleae (Fig. 4, Supplementary Figs. 4–5, Movie 4). Furthermore, we discovered a different mineralized structure in the proximal ST, which was previously described as a part of the SSL^42,59,63,64^. In this paper, we refer to the newly-distinguished structure as the round window arch (RWA); this term was inspired by its arch-shaped 3D form and anatomical position along the upper rim of the RWM (Fig. 4, Movie 4). Similar to the RWM itself, the shape of the RWA were symmetrical within the same subject (Supplementary Fig. 4).

These findings illustrate once again the importance of simultaneous, nondestructive 3D visualization of soft and mineralized structures, as could be achieved with CECT. On the one hand, clear visualization of the mineralized structures enabled us to differentiate porous bone of the SSL from the dense, cortical bone of the RWA in the most proximal region of the cochlea (Fig. 4). On the other hand, 3D rendering of these bony structures in relation to the soft tissues allowed us to easily identify the SSL in the deeper cochlear regions along with the SL, and discern it from the RWA, which follows the curvature of the RWM (Movie 4). Finally, identification of these structures on CECT images of a gerbil cochlea, and their distinct composition on previously published confocal immunohistochemistry images of the human cochlea (elastin fibers are present in the SSL, but not in the bone of the RWA)^42^ (Supplementary Fig. 6), confirmed our conclusion that the SSL and the RWA are two different structures.

### Electrode insertion trauma visualized and quantified in 3D

Since we established that CECT provides clear nondestructive 3D visualization of the intracochlear structures, in the last step we investigated whether this technique can also be used to directly assess CI-related insertion trauma in 3D.

**Figure 5:**
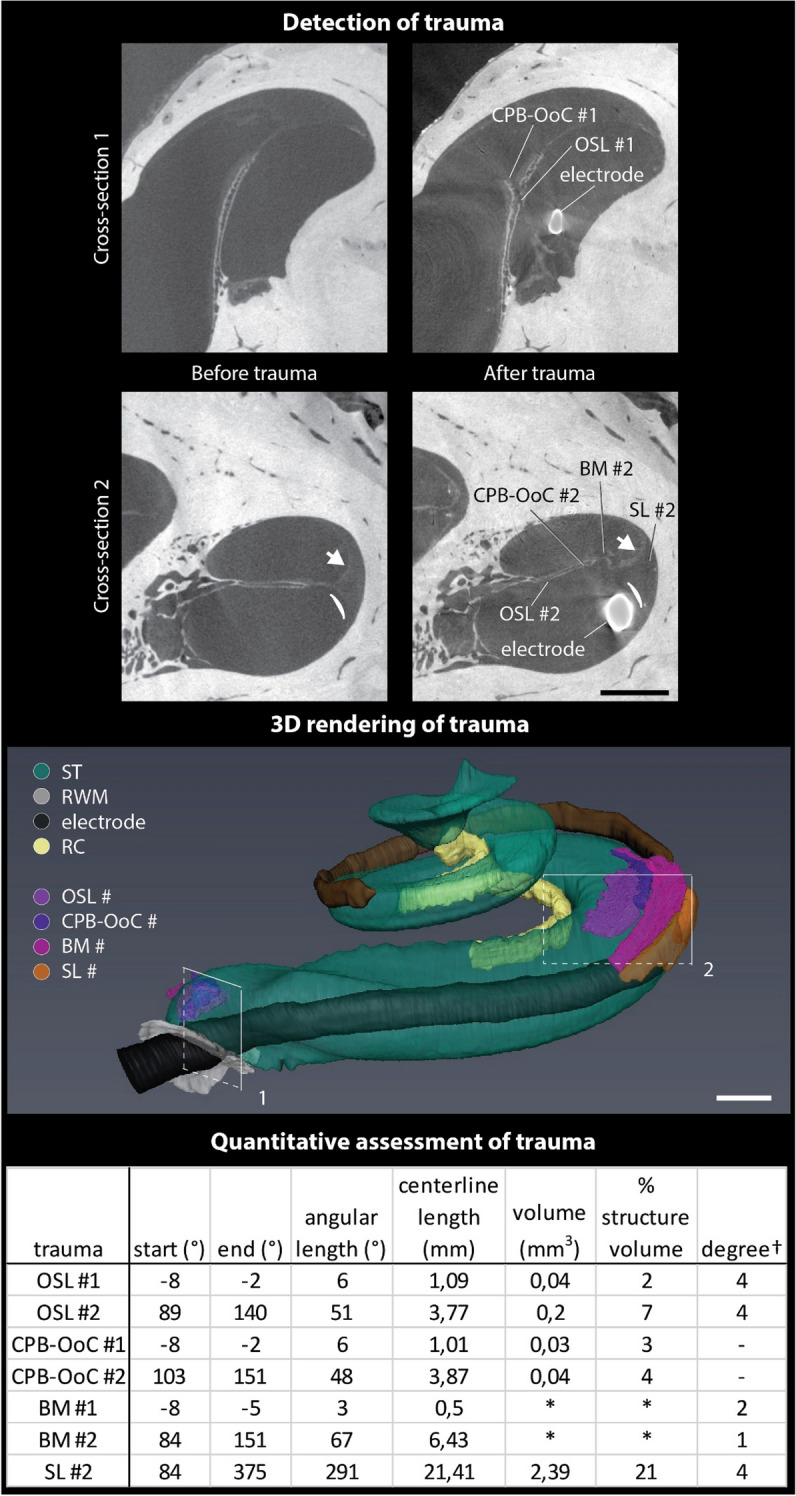
Detection, 3D rendering and quantitative assessment of electrode insertion trauma in IE_4R. *(top)* The representative CECT cross-sections of the traumatized regions 1 and 2, before and after trauma. Cross-section 1 after trauma, shows a fracture of the OSL and a tear in the OoC. Cross-section 2 after trauma, shows a fracture of the OSL; a deformation of the BM and the CPB; a tear of the SL in the ST (white curved line); and a deformation of the SL in the scala vestibuli (white arrow). *(middle)* 3D rendering of inserted straight electrode within the ST together with the traumatized parts of the intracochlear structures (OSL #, CPB-OoC #, BM #, SL #). The position of the cross-sections is indicated on the 3D rendering. *(bottom)* Overview of the trauma characteristics in each separate intracochlear structure, with its angular position and dimensions, centerline length and volume. % structure volume indicates the percentage of original, pre-insertion structure volume, which has been affected by trauma. For the BM, the exact volumes could not be determined (*), since the resolution of the segmented data (18.9 µm^3^) was not sufficiently high, compared to the thickness of the BM (25 µm on the original scan at 6.3 µm). In the last column, each trauma was also graded, in accordance to the scale of Eshraghi et al. (†), as described in the “Methods” section^15^. BM: basilar membrane; CPB-OoC: cochlear partition bridge—organ of Corti complex; OSL: osseous spiral lamina; RC: Rosenthal’s canal; RWM: round window membrane; SL: spiral ligament; ST: scala tympani. Scale bar: 1 mm.

CECT data acquisition before and after electrode insertions in the cochlea of IE_4R enabled us to accurately detect morphological changes of both mineralized and soft intracochlear structures, which are characteristic for electrode insertion trauma^15,16,19^. Subsequent segmentation and 3D analysis also allowed determination of the electrode insertion depth (380°) and standardized, quantitative description of trauma to separate intracochlear structures (Fig. 5). The conventional trauma grading scale of Eshraghi et al.^15^ was additionally used to demonstrate the compatibility and the precision of CECT-based trauma characterization. Finally, 3D rendering of these data gave insights into the possible mechanisms of trauma (Movie 5). In particular, the origin of the SL trauma could most likely be attributed to the last electrode insertion, since its 3D shape perfectly matched the contour of the electrode, which transgressed from the ST into the SL.

## Discussion

In this study, we introduced and validated Hf-WD POM-based CECT for simultaneous 3D visualization of the bony and soft intracochlear structures in human cochleae. Contrary to the previous studies, the fresh-frozen human cochleae were imaged nondestructively for the first time, without removal of the intracochlear fluid, fixation of the samples or usage of destructive CESA’s. In addition, it is possible that Hf-WD POM also exhibits antimicrobial properties, similar to other polyoxometalate compounds, which could further improve tissue preservation over time^65^. This is extremely important, since fresh and fresh-frozen cochleae are the best model for a living cochlea^40,41^.

CECT-based segmentation and simultaneous 3D analysis of the soft and mineralized intracochlear structures led to important discoveries in the microanatomy of the human cochlea: it allowed us to locate the apical bulb of the RC in the third cochlear turn, to measure in 3D the dimensions of the separate intracochlear structures, to determine the true thickness of the RWM, to detect the SSL beyond the proximal cochlear region and to discover the round window arch (RWA) along the upper rim of the RWM. Furthermore, we demonstrated that in addition to the anatomical characterization of the intracochlear structures, Hf-WD POM-based CECT could also be used for nondestructive, quantitative assessment of electrode insertion trauma in 3D.

Reliable, simultaneous visualization of mineralized and soft-tissue intracochlear structures in 3D is important to gain profound understanding into the microanatomy of the human cochlea. The dimensions and the 3D geometry of the intracochlear structures and compartments, such as the RWM, the ST and the RC are not only interesting from a fundamental perspective of anatomical study, but also lie at the basis of new electrode design^66^ and development of atraumatic surgical techniques^67^ for CI surgery. Our findings, demonstrating the ability of CECT to visualize and quantitively characterize these structures, form an important contribution to the field. While CECT imaging cannot be performed on living patients, the acquired microanatomical knowledge can complement current clinical imaging techniques with limited resolution by building an imaging atlas for individualized therapy design^68,69^ and by correlating microanatomical features to clinically measurable parameters, such as the width of the first cochlear turn^55^. Furthermore, CECT can act as a reliable reference—as opposed to classical histology—for novel high-resolution imaging techniques which are currently in full development for application in clinical otology, such as optical coherence tomography^70^. CECT also has important benefits over more recent alternatives, such as synchrotron radiation phase-contrast imaging (SR-PCI)^12,42,49,71^ and thin-sheet laser imaging microscopy (TSLIM)^72^. TSLIM imaging requires very extensive tissue preparation with fixation and decalcification, similar to classical histology, which is destructive for intracochlear tissues. SR-PCI can only clearly visualize the edges of the soft tissues, and their contrast is similar to the mineralized structures, which can complicate anatomical analysis and segmentation of the imaging data. Furthermore, microCT imaging technology is much more accessible than SR-PCI.

When analyzing the 3D anatomy of the intracochlear structures, special attention was given to the SSL, which has been recently mentioned in the context of proximal cochlear trauma in CI surgery^12,42^. Our discovery that the SSL is present throughout the full first cochlear turn implies that this outer wall structure can not only be traumatized adjacent to the RWM^12,42^, but also in the deeper cochlear regions. As mentioned earlier, trauma of the cochlear outer wall causes fibrosis of the cochlear compartments in some patients and additional ossification in the others^4,16,18,73^, whereby primarily intracochlear ossification has been associated with a worse hearing outcome in CI patients^4,16,73^. The underlying pathophysiology of ossification remains unclear, but it could be triggered by the presence of loose bone debris, resulting from trauma of bony intracochlear structures^4^. Following this line of thought and based on the newly-discovered prominent presence of the SSL in the outer wall of the human cochlea, it is plausible to hypothesize that fibrosis would occur when only the soft-tissue SL was traumatized, whereas ossification would be triggered when there was additional trauma of the bony SSL. Furthermore, the newly-found presence of the SSL in the high- and mid-frequency regions of human cochlea, in accordance to the frequency maps of the BM^47,48,74^, strongly indicates its functional involvement in the human hearing, rather than being a merely rudimentary structure. Previous studies postulated that the SSL increases the stiffness of the BM and could contribute to high-frequency hearing—either directly mechanically or, evolutionary, through impedance matching of the mammalian cochlea to a stiffer middle ear^42,50,75–81^.

Meticulous analysis of the SSL anatomy also led to the discovery of the RWA, a bony structure which is really only present along the upper rim of the RWM. The functional role of the RWA inside a human cochlea remains to be determined in future studies: it could be a rudiment from embryological development of the cochlea, it could also act as a mechanical shield between the BM and the RWM, which was originally hypothesized as a function of the SSL by Agrawal et al.^42^.

Finally, we were able to demonstrate that CECT enables detection and quantitative characterization of electrode insertion trauma in 3D. Assessment of intracochlear trauma is a crucial step in preclinical electrode insertion studies, which are striving to unravel the mechanics of insertion, as well as develop and test novel techniques for atraumatic CI surgery. In these studies, electrode insertion trauma is usually evaluated by means of classical histological analysis^15,19,20,40^. However, this method has limitations: destructive tissue preparation can lead to false positive outcomes, evaluation of 2D slices is prone to false negatives, and the analysis also cannot be repeated more than once due to the destructive nature of histopathological processing. With CECT, we were able not only to visualize traumatized bony and soft intracochlear structures simultaneously with the inserted electrode, but also to compare post-insertion to the pre-insertion imaging data. Such approach for nondestructive 3D assessment of electrode insertion trauma in relation to the inserted electrode array is not only more reliable, but can provide additional insights into the insertion mechanics. Furthermore, this method opens new possibilities for repeated analysis in the same sample, which can reduce the need for human cadaveric tissue and enable the study of electrode insertion versus extraction trauma. Contrary to standard microCT imaging^28,31^, CECT enables clear visualization of cochlear soft tissues structures in their original shape, without the need for removal of the intracochlear fluid, which enables more accurate assessment of soft-tissue electrode insertion trauma. In addition, CECT imaging of the human cochlea has the potential to enable highly accurate modelling and testing of novel techniques for atraumatic electrode insertion, such as impedance measurements^82,83^ and robotics-assisted insertion^31^. Finally, CECT holds a great promise for nondestructive, quantitative 3D study of cochlear pathologies, underlying hearing loss, which are currently mainly being investigated with classical histology^84^.

Hf-WD POM-based CECT imaging technology does have some inherent limitations. The primary challenge with staining human cochleae is that soft tissue contrast may vary amongst different samples. In fixed samples, higher contrast can be achieved through long staining times^37^, but prolonged incubation times could compromise tissue integrity in fresh cochleae. Based on our first experience, intracochlear diffusion of the CESA into fresh cochleae can be optimized by obtaining good exposure of the RWM and other accessory structures for contrast entry (the stapes and the modiolus), and by avoiding intracochlear air bubbles. Another limitation is that Hf-WD POM compound can precipitate during the staining period. In our study, intracochlear precipitation of Hf-WD POM could be tackled by placing the sample on a shaker plate during the staining period. This approach furthermore accelerates intracochlear CESA diffusion and counteracts base-to-apex concentration gradient^44^. Generally speaking, the resolution of microCT technology is lower than classical histology. However, subcellular visualization of the tissues has previously be achieved with X-ray nanocomputed tomography (nanoCT)^85^, which can be explored for intracochlear imaging in future studies. Finally, on the cochlear CECT images with an inserted electrode, the metal artifacts were corrected by replacing pixels affected by high metal attenuation with interpolated values from the neighboring pixels using simple projection completion approach. This approach could interfere with detection small trauma, limited to the regions, which were severely affected by metal artifacts. In the future, metal artifact reduction can be further improved by using more sophisticated algorithms, such as reprojection or iterative reconstruction^86^.

To summarize, this study introduced and validated Hf-WD POM-based, high-resolution CECT for non-destructive, 3D imaging of fresh human cadaveric cochlea. With CECT, we obtained unprecedented simultaneous visualization of the mineralized and soft intracochlear structures, which allowed us to analyze their true dimensions, 3D course and integrity, and to reveal previously unknown aspects of the human cochlear microanatomy. These findings incite further research into the functional anatomy of the human cochlea, and set the stage for intracochlear X-ray-based 3D histology as a promising tool for anatomopathological study of the human cochlea and for preclinical electrode insertion studies.

## Methods

### Human cochleae

Two left-sided (IE_1L, IE_2L) and two right-sided (IE_3R, IE_4R) fresh-frozen human cadaveric inner ears were used in this study, whereby the samples IE_2L and IE_3R originated from the same subject (Supplementary Fig. 1, Supplementary Table 3). All four temporal bones were harvested within 72 h post mortem from individuals who underwent a clinical brain autopsy at the University Hospitals of Leuven. Informed consent was obtained from all subjects, their next of kin or legal guardian(s). Harvesting and use of the temporal bones was conducted in accordance with the Helsinki declaration and approved by the Medical Ethics Committee of the University Hospitals of Leuven (S65502). To minimize the required amounts of the CESA and to facilitate its diffusion into the cochlea, the inner ears (approx. 10 mm × 10 mm × 20 mm) were dissected out of the temporal bones^63^. After the surgical dissection, the isolated inner ears were thoroughly evaluated under a surgical microscope, to exclude obvious anatomical malformations and trauma to the stapes, the RWM, the cochlear capsule and the semicircular canals of the vestibular system. In addition, the stapes mobility and the integrity of the RWM were visually evaluated under the surgical microscope by confirming movement of the RWM, without leakage of the intracochlear fluid, when gentle pressure is applied on the stapes. No pathological findings were detected during the inspection and dissection of the temporal bones. The samples IE_1L and IE_4R were frozen twice: once before and once after the dissection; IE_2L and IE_3R were frozen once, after the dissection. The samples were not fixed or decalcified. On the evening before the staining experiment, the fresh-frozen samples were thawed overnight at 4 °C.

### CECT—staining, image acquisition and image reconstruction

The CESA used in this study was an in-house synthesized Hf-WD POM^36^. To normalize CECT data of different days for each sample, a small piece of parafilm foil was wrapped around the samples as a reference material. The isolated inner ears were submersed in 10 ml Hf-WD POM in phosphate-buffered saline solution (35 mg/ml) for up to 7 days, while placed on a horizontal shaker plate at 4 °C to accelerate CESA diffusion through the cochlear lumen and minimize base-to-apex concentration gradient. 35 mg/ml solution was previously demonstrated to be the lowest possible concentration for effective staining of the different soft tissues^36^. The stained cochleae were imaged using a Phoenix Nanotom M (GE Sensing & Inspection Technologies GmbH, Wunstorf, Germany), equipped with a tungsten target, at 6.3 µm^3^ isotropic voxel size. 2400 frames were acquired over 360° (Supplementary Table 4). The cochlea IE_4R was imaged prior to electrode insertion and subsequently—after a total of six insertions using a precurved electrode (Nucleus® CI532/CI632; Cochlear Ltd., Sydney, Australia) and two straight electrodes (Nucleus® CI422/522; Cochlear Ltd., Sydney, Australia and HiFocus™ SlimJ, Advanced Bionics AG, Stäfa, Switzerland)—with a straight 23 mm long HiFocus™ SlimJ electrode array inside the cochlea. The last four insertions were performed in a traumatic manner: under suboptimal insertion vector^87^ and characterized by an increased surgical-mechanical resistance. More than one traumatic insertion was performed to detect different types of electrode insertion trauma in a single post-insertion CECT dataset. The samples TB_2L and TB_3R were imaged at a long and a short detector distance to determine which settings give the best visualization of the intracochlear structures. The data were reconstructed in Datos|x (GE Measurement and Control solutions), while applying scan optimization (projection filter, inline volume filter, and beam hardening correction) and exported as 16-bit TIFF slices.

### CECT—data analysis and display

The 16-bit slices (.tiff) were converted 8-bit slices (.jpg) using an in-house developed MatLab tool, which simultaneously windowed the histogram range to the dynamic range of the dataset and normalized the data to the last Hf-WD POM dataset^39^. In the normalization step, the grey values were matched between datasets for the two reference materials: the cortical bone of the cochlear capsule and the parafilm foil. After that, all data were cropped to the cochlear region in CTAn (Bruker MicroCT, Kontich, Belgium) and reoriented in accordance with the cochlear coordinate system^88^ in DataViewer (Bruker MicroCT, Kontich, Belgium). The cochlear coordinate system is a 3D, cylindrical coordinate system with the following references: the plane of rotation goes through the basal turn of the cochlea and is perpendicular to the modiolus; the rotation axis goes through the center of the modiolus; the 0° reference angle is the center of the round window (here, the RWM).

The visualization of the intracochlear soft tissues in consequence of Hf-WD POM absorption was assessed in DataViewer. For this purpose, the visualization of soft tissue structures (BM, SL, OoC, RM) in each of the three cochlear turns was graded immediately before and after the submersion in Hf-WD POM, as well as on day 1, 2, 3, 5 and 7 of staining (Fig. 1, Supplementary Fig. 2). To avoid bias, the datasets were graded in a non-chronological order. IE_4R was only imaged on day 3 and day 5. IE_3R could not be imaged on day 5, due to a technical problem with the microCT device and the dataset of day 7 was excluded from the contrast absorption analysis, due to a significantly lower signal-to-noise ratio (p-value < 0.05)—assessed as the contrast between reference materials^89^, compared to the datasets of the previous days—which interfered with the visualization of the soft tissue structures.

The overall soft tissue visualization in IE_2L was worse than in the other samples (Supplementary Fig. 2). On the one hand, this difference could be attributed to the fact that IE_2L contained the tympanic membrane (Supplementary Fig. 1), which could have negatively affected the free, non-turbulent flow of the CESA solution around the RWM during the staining experiment. On the other hand, IE_2L was the only sample, containing air bubbles, which could have interfered with Hf-WD POM diffusion through the intracochlear fluid within the cochlea.

The dataset with the best visibility of the soft tissue structures in each sample was selected for segmentation and further 3D analysis: short detector distance dataset of day 7 for IE_1L and IE_2L, day 3 for IE_3R and day 5 for IE_4R. In particular, the visibility of the Reissner’s membrane typically further improved after day 3, which is important for the separate segmentation of the scala media and the scala vestibuli compartments. On the data of IE_4R with electrode, the metal artefacts were suppressed by applying a simple projection completion approach using linear interpolation^90^. For that purpose, an initial reconstruction is made with the Feldkamp-Davis-Kress (FDK) algorithm^91^. In that reconstruction, the metals are segmented using thresholding. The resulting binary metal image is forward projected to identify in the projections all pixels that have been affected by high metal attenuation. The values of those pixels are replaced with interpolated values, computed from the neighboring pixel values that are not affected by metals. The final image is computed from the corrected projections with FDK. As discussed e.g. by Gjesteby et al.^86^, several improvements to this algorithm and numerous alternative metal artefact reduction approaches have been proposed. However, many of those involve a reprojection or even iterative reconstruction, which could not be applied here because the field of view only covered a part of the object. Although further improvement may be achievable with more sophisticated algorithms, the simple projection completion approach was found to be effective for the problem at hand.

All the selected datasets were resized to an isometric voxel size of 18.9 µm^3^ in CTAn and manually segmented in Avizo (FEI Visualization Sciences Group, Thermo Fisher Scientific Inc., Bordeaux, France). The operator-dependency of the manual segmentation was evaluated by measuring the RWM volume, based on the results of two experienced and two non-experienced operators (Supplementary Table 5), and appeared to be negligible for the experienced operators.

The cochlear width, length and height^9^ were measured manually in DataViewer. The number of cochlear turns, together with the angular measurements were done manually on the segmented data in Avizo. All manual measurements were carried out twice, and the averaged values were included in the paper. The volume and centerline length of the cochlear structures were automatically determined on the segmented data in Avizo. The thickness of the RWM was calculated automatically in CTAn, using a sphere-fitting algorithm^92^ on the segmented data, in steps of 2 voxels. After that, the resulting thickness map was visualized in Avizo. In IE_4R, the electrode position and electrode insertion trauma were assessed in Avizo on the segmented data. The percentage of traumatized structure was calculated with respect to the structure volume before the electrode insertion. As a reference, the degree of trauma was quantified in accordance to the conventional scale by Eshraghi et al.^15^: 0, no observable trauma; (1) elevation of the BM; (2) rupture of BM; (3) electrode in the scala vestibuli; and (4) severe trauma such as fracture of the OSL or modiolus, or tear of stria vascularis within the SL.

Graph display and statistics were done in Graphpad Prism 9.3.1 (Graphpad Software, San Diego, California, USA). The measurements are presented as measured values and ranges. The 3D renderings were created in Avizo and CTvox (Bruker MicroCT, Kontich, Belgium). In Fig. 4, the CECT images were filtered in Avizo, using non-local means, followed by unsharp-masking, to accentuate soft tissue visualization; no filtering was applied on the other provided CECT images. Figures and movies were finalized using Adobe Illustrator and Adobe Premiere Pro respectively.

### Classical histology

Histological analysis of the samples IE_2L and IE_3R was performed after the CECT study. No histology was performed in IE_1L and IE_4R, because these samples were damaged after the experiments. Prior to histological processing, the ST and the scala vestibuli were fenestrated with a surgical drill at approximately 180° (Movie 1), to make the inner ear accessible for fixation and embedding agents, while preserving the microanatomy around the RWM as good as possible. The samples were fixed in a 4% formaldehyde solution for 5 days, followed by dehydration in ethanol 50% and 70%. Subsequently, the cochleae were imaged using CECT (Supplementary Table 4) to assess for possible trauma and to guide the position of the histological sections—one transbasal and one transmodiolar section was produced in each sample (Movie 1). The CECT datasets demonstrated collapse of the scala media compartment in the parts without intracochlear fluid and trauma to the OSL, the SL and the BM in close proximity to the regions where the cochleae were opened (Supplementary Fig. 3, CECT open cochlea). LLS Rowiak (LaserLabSolutions, Germany) performed polymethylmethacrylate embedding, optical coherence tomography guided sectioning with a laser microtome TissueSurgeon^93^ and staining of the slices with Masson–Goldner and Von Kossa/van Gieson.

### Validation of CECT images

For the qualitative validation, the CECT datasets were reoriented at the same cutting angle as the subsequent histological sections using DataViewer (Bruker MicroCT, Kontich, Belgium) and the precise interpolation of the matching CECT image was found using an in-house developed MatLab tool^94^. The four histological slices obtained from two samples were found to be sufficient to evaluate the qualitative resemblance between CECT and histological data. Quantitative validation was performed by comparing the dimensions of the entire mineralized cochleae, which are not affected by histological tissue processing techniques, to previous reports: the width, length and height of the entire cochlear spiral, the number of cochlear turns and the total volume of the cochlea.

## Supplementary Information

Supplementary Information 1.

Supplementary Video 1.

Supplementary Video 2.

Supplementary Video 3.

Supplementary Video 4.

Supplementary Video 5.

## Data Availability

All data analyzed during this study are included in this published article. The full CECT datasets can be provided by Nicolas Verhaert (nicolas.verhaert@kuleuven.be) upon reasonable request.
