# Supplementary material for: Human cochlear microstructures at risk of electrode insertion trauma, elucidated in 3D with contrast-enhanced microCT
Source: Sci Rep. 2023 Feb 7;13:2191. doi: 10.1038/s41598-023-29401-6 (PMC9905077; doi:10.1038/s41598-023-29401-6)
Supplement: Supplementary file 1 — Supplementary Information 1. [file 41598_2023_29401_MOESM1_ESM.docx]

**Supplementary information for**

**Human cochlear microstructures at risk of electrode insertion trauma, elucidated in 3D with contrast-enhanced microCT**

*Anastasiya Starovoyt ^a, b^ , Grzegorz Pyka ^c^ , Tristan Putzeys ^a, b, d^ , Tim Balcaen ^c, e, f^ , Jan Wouters ^a, b^ , Greet Kerckhofs ^c, f, g, h,§^ , Nicolas Verhaert ^a, b, i, *, §^*

Author affiliations:

1. ExpORL, Department of Neurosciences, Katholieke Universiteit (KU) Leuven, 3000 Leuven, Belgium
2. Leuven Brain Institute, Department of Neurosciences, KU Leuven, 3000 Leuven, Belgium
3. Biomechanics Laboratory, Institute of Mechanics, Materials, and Civil Engineering, Université Catholique de (UC) Louvain, 1348 Louvain-la-Neuve, Belgium
4. Laboratory for Soft Matter and Biophysics, Department of Physics and Astronomy, KU Leuven, 3000 Leuven, Belgium
5. Molecular Design & Synthesis, Department of Chemistry, KU Leuven, 3001 Leuven, Belgium
6. Prometheus, Division of Skeletal Tissue Engineering, KU Leuven, 3000 Leuven, Belgium
7. Department of Materials Science and Engineering, KU Leuven, 3000 Leuven, Belgium
8. Institute of Experimental and Clinical Research, UC Louvain, 1200 Woluwé-Saint-Lambert, Belgium
9. Dept. Otorhinolaryngology, Head and Neck Surgery, University Hospitals of Leuven, Leuven, 3000 Belgium

* Corresponding author: Nicolas Verhaert (email: [nicolas.verhaert@kuleuven.be](mailto:nicolas.verhaert@kuleuven.be))

*§* G.K. and N. V. contributed equally to this work and share last authorship.

**SUPPLEMENTARY FIGURES**

**
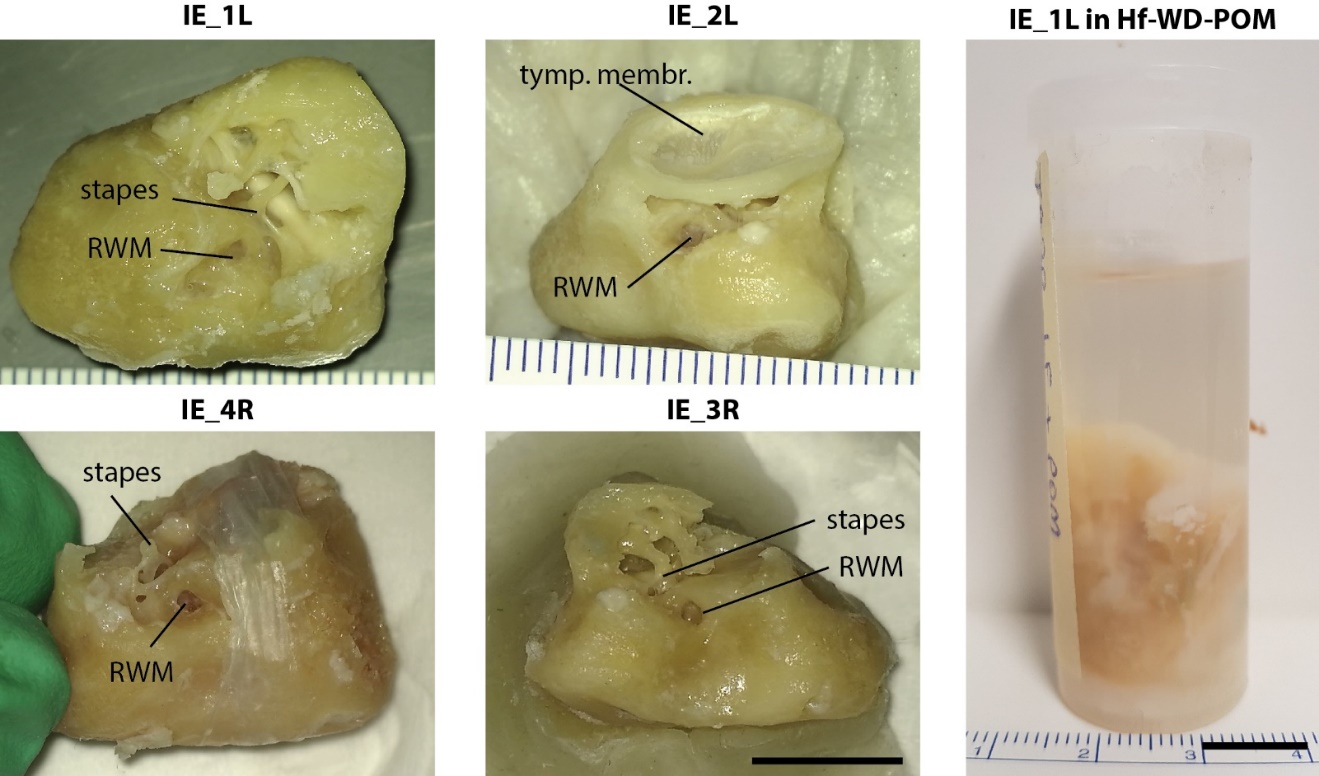
**

**Supplementary Figure 1. Overview of the four fresh-frozen inner ear samples.** The stapes, the RWM and the tympanic membrane (tymp. membr., only present in IE_2L) are indicated for the orientation. For IE_4R, the position of the paraffin foil, used for image normalization, is shown (described in the Methods). The figure on the right depicts IE_1L in Hf-WD POM staining solution. The photographs were taken from ‘inferior view’ according to ***Figure 3***. *RWM: round window membrane*. Scale bar: 10 mm.


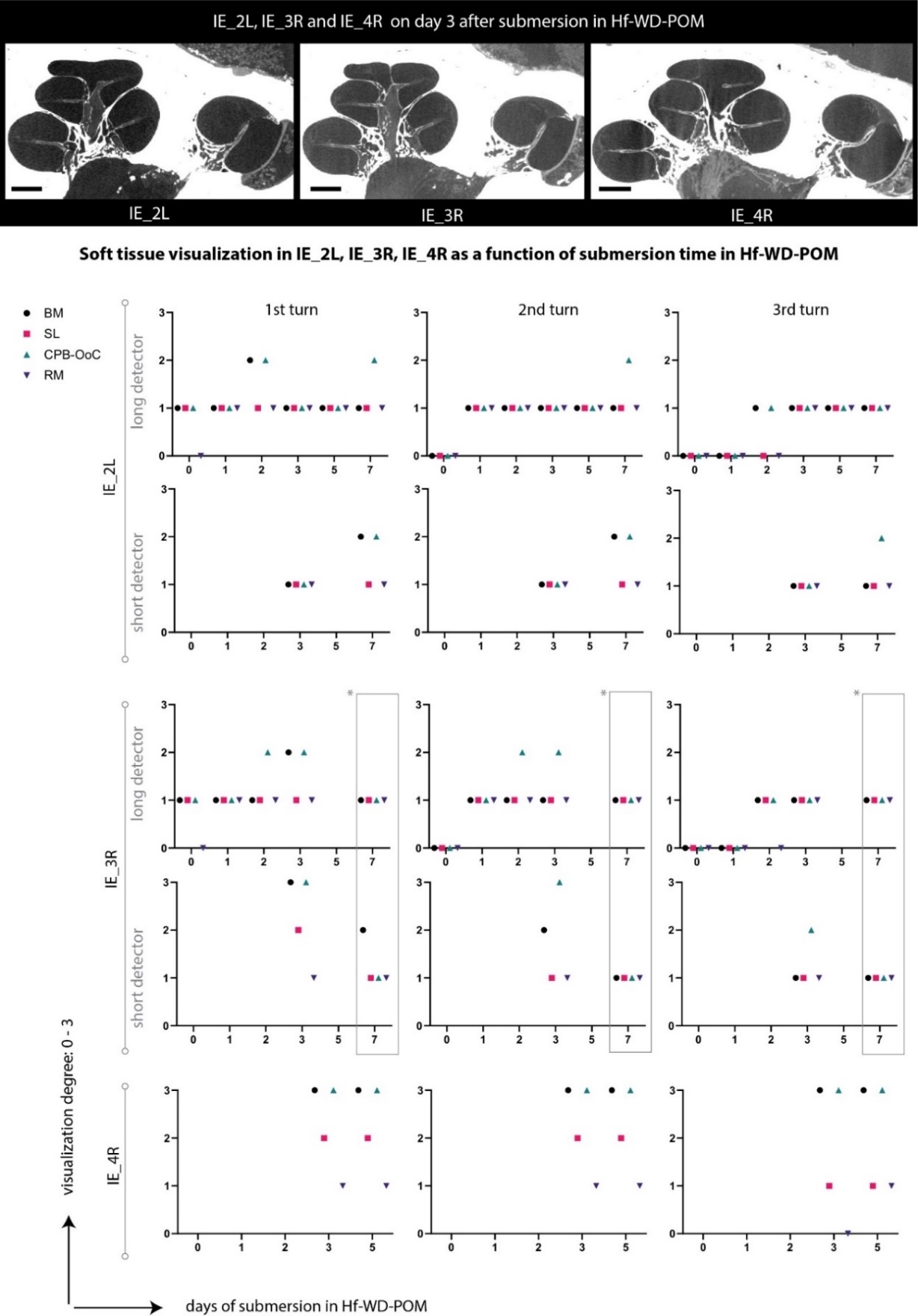


**Supplementary Figure 2. Hf-WD POM-related soft tissue visualization in IE_2L, IE_3R, IE_4R.** IE_2L and IE_3R were imaged at a long and short detector distance, as discussed in the Methods section. Soft tissue visualization was graded as follows: 0, not visible; 1, partially visible on some slices; 2, partially visible on all slices; 3: entirely visible on all slices. In IE_2L, soft tissue visualization was worse than in the other samples, which is discussed in the Methods section. In IE_3R, the results of day 7 (indicated with *) were excluded from visualization analysis, due to a significantly lower signal-to-noise ratio (p-value < 0.05), compared to the datasets of the previous days.


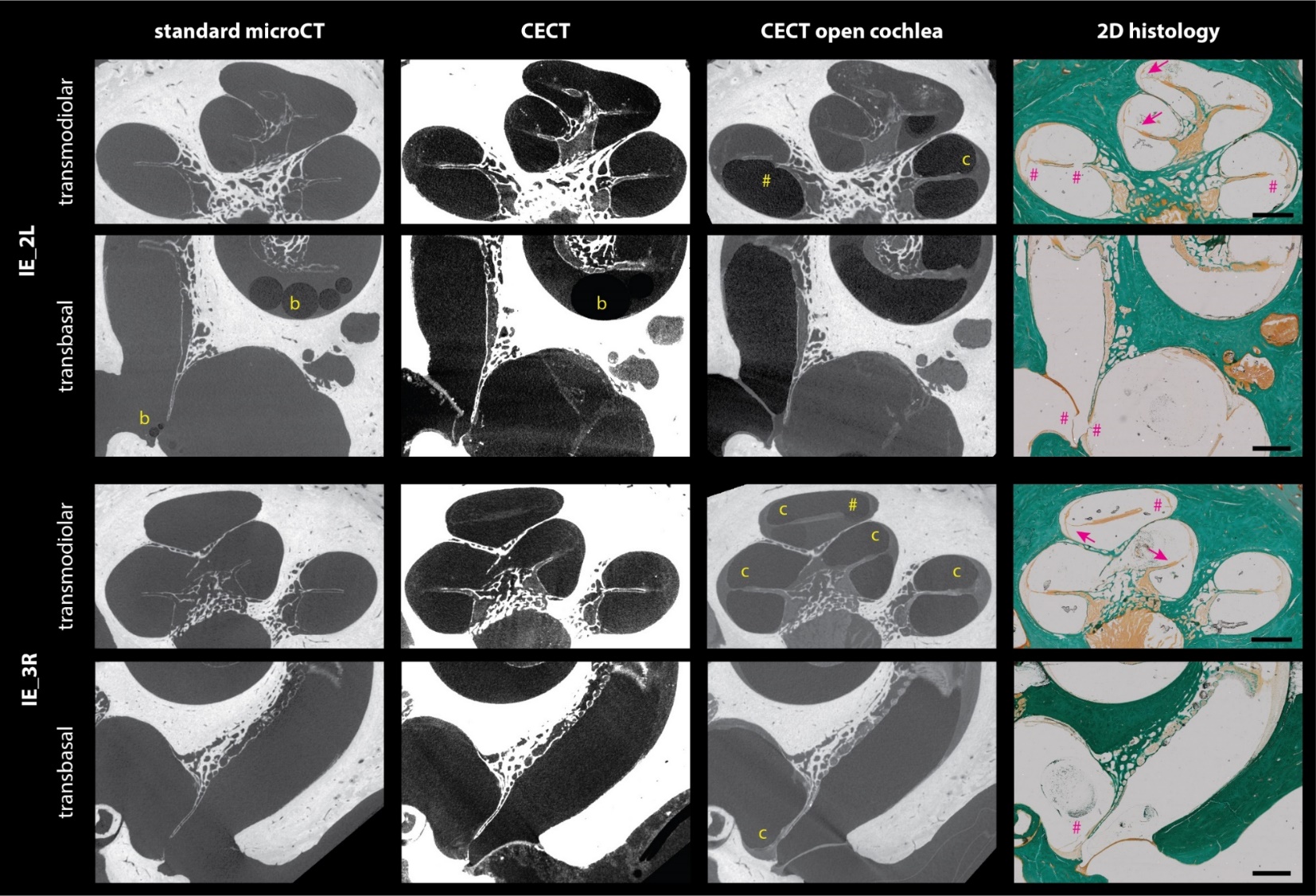


**Supplementary Figure 3. Comparison of CECT and istological cross-sections in IE_2L and IE_3R.** The microCT sections were matched to the corresponding 2D histological section, using the Matlab-tool of Kerckhofs et al. (G Kerckhofs et al., 2008) The visualization of air-bubbles (b) in IE_2L, collapse of the scala media (c) and intracochlear trauma (#) related to the opening of the cochleae and/or histological processing are indicated on the images. The deformations of the delicate intracochlear structures on histological slices are indicated with arrows. The Reissner’s membrane was torn or absent on all histological preparations (not specifically indicated on the images). Scale bar: 1 mm.


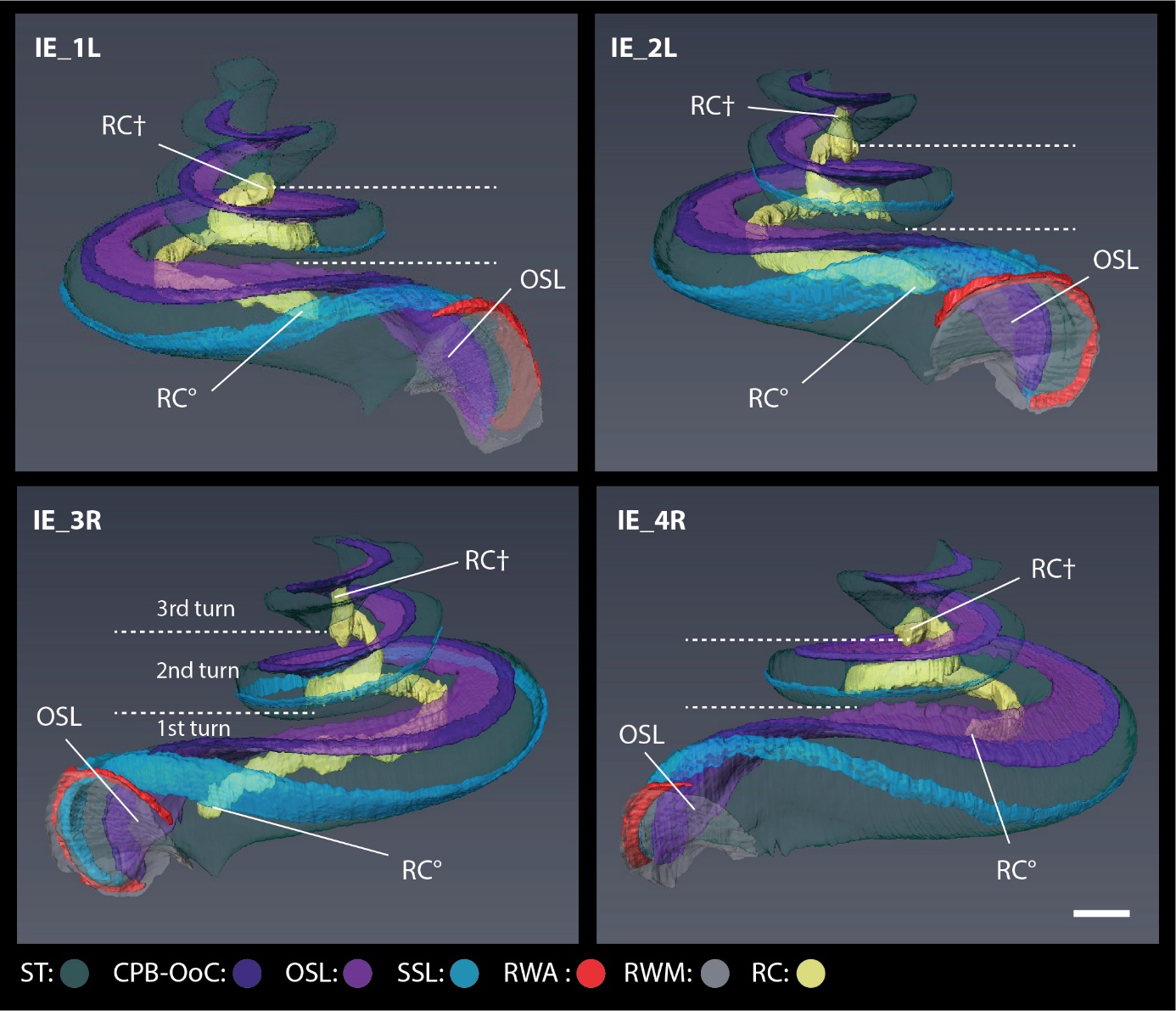


**Supplementary Figure 4. CECT-based 3D rendering of the ST, the RC, the SSL and the RWA in the four cochleae.** The proximal (RC°) and the distal end of the RC (RC†) are indicated together with the OSL. The apical bulb of the RC is smaller in IE_1L and IE_4R, but it is nevertheless positioned adjacent to the ST of the third cochlear half-turn. Note that the short RWA follows the upper margin of the RWM, unlike the longer SSL, which extends into the second cochlear turn, following the curvature of the ST together with the SL (not visible here, but shown in ***Movie 4***). *CPB-OoC: cochlear partition bridge – organ of Corti complex; OSL: osseous spiral lamina; RC: Rosenthal’s canal; RWA: round window arch; RWM: round window membrane; SSL: secondary spiral lamina; ST: scala tympani.* Scale bar: 1 mm.


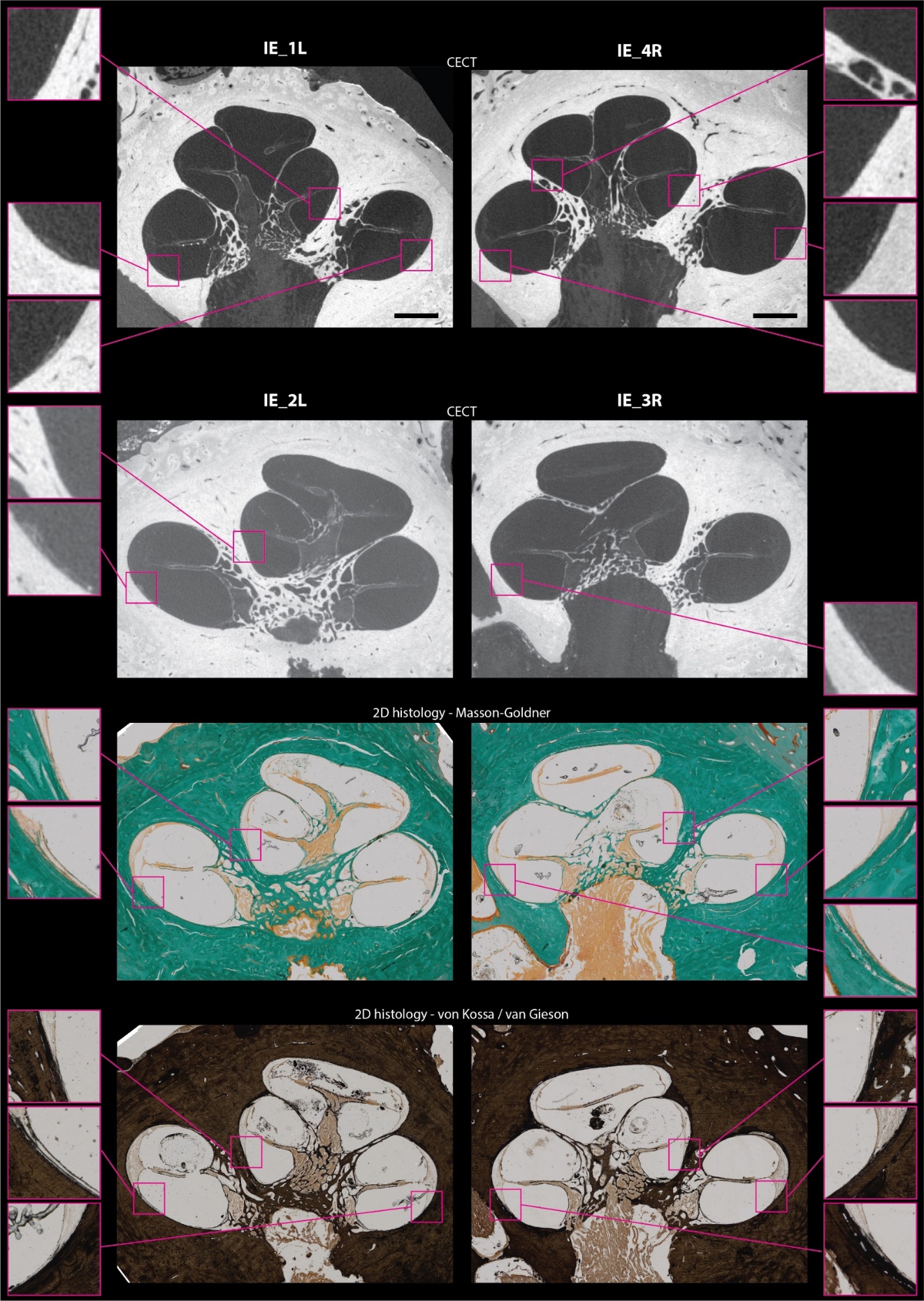


**Supplementary Figure 5. The presence of the SSL beyond the proximal area in the four cochleae.** Due to its porous structure and small size, some parts of the SSL are not always visible on each CECT slice. Note that the SSL is stained as bone on classical histology (blue on Masson-Goldner, dark-brown on von Kossa/van Gieson). Scale bar: 1 mm.

**
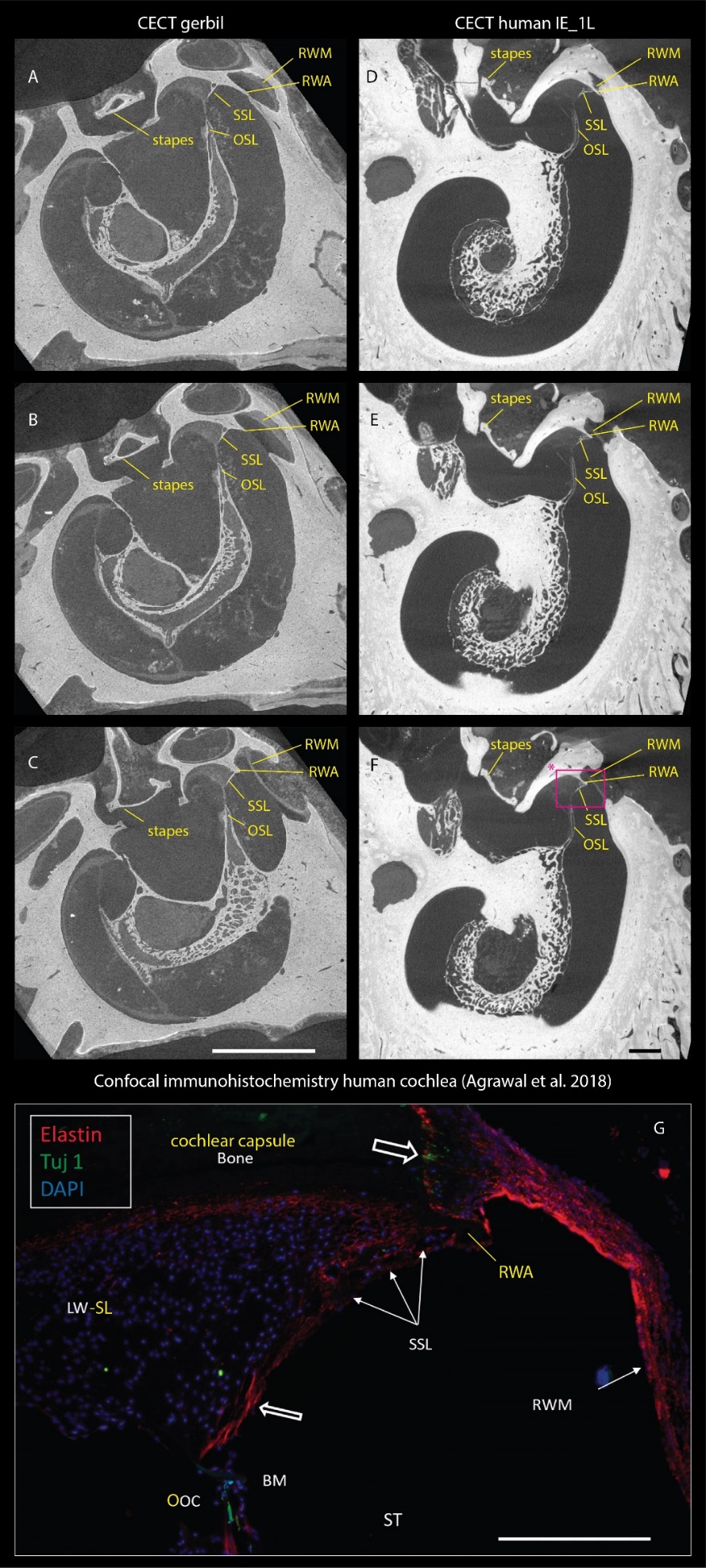
Supplementary Figure 6. The SSL versus the RWA in a gerbil and a human cochlea.** The CECT images A – C correspond to a gerbil cochlea; the CECT images D – G correspond to a human cochlea. The *SSL* and the *RWA* are indicated with respect to the other intracochlear structures. The *RWA* is more prominent and distinctive from the *SSL* in a gerbil cochlea, compared to the human cochlea. The rectangle (*) in F indicates the approximate region, depicted on the confocal immunohistochemistry image in G, which was adapted from Agrawal *et al.* 2018^1^, published under a Creative Commons CC-BY 4.0 license. Additional inscriptions were added in the yellow color, to match the rest of this manuscript. Note, that contrary to the *SSL*, there appears to be no elastin expression in the *RWA*. *BM: basilar membrane; LW-SL: lateral wall – spiral ligament; OoC: organ of Corti; OSL: osseous spiral lamina; RWA: round window arch; RWM: round window membrane; SSL: secondary spiral lamina, ST: scala tympani.*

Scale bar in A – F: 1 mm. Scale bar in G: 100 µm.

**SUPPLEMENTARY TABLES**

**Supplementary Table 1 (next page). Overview of the cochlear dimensions in the four cochleae.** Cochlear width, length and height were measured as previously described by Shin et al.^2^. The scala vestibuli and the scala media could not be segmented separately in IE_4R, which explains their missing values in that sample. The total cochlear volume was calculated as the volume sum of the RWM, cochlear compartments, CPB-OoC, OSL, SL and SSL. The parameters which follow the same trend as the cochlear width, based on their available values, are indicated with *. The cochlear dimensions, which appeared symmetrical within the same subject are indicated with **. Note that the measured length of scala media and the ST based on CECT images, is larger than previously assessed on histology (for scala media) or clinical CT and magnetic resonance imaging (MRI) imaging data (for ST). This is due to the fact that dimensional measurements on 2D histological slices are prone to underestimation in consequence of shortening error^3^ and tissue shrinkage^3–7^, while the limited resolution of clinical imaging modalities impedes precise segmentation of the ST and the scala vestibuli in the apical transition zone.

*CPB-OoC: cochlear partition bridge – organ of Corti complex; OSL: osseous spiral lamina; RC: Rosenthal’s canal; RC-bulb: the angular position of the transition between the spiraling part of the RC and the apical bulb; RC-prox.: the angular position of the proximal end of the RC; RWA: round window arch; RWM: round window membrane; SL: spiral ligament; SM: scala media; SSL: secondary spiral lamina; SSL-dist.: the angular position of the distal end of the SSL; ST: scala tympani; SV: scala vestibuli.*

|  | **IE_1L** | **IE_2L** | **IE_3R** | **IE_4R** | **measured range** | **range in the literature** |
| --- | --- | --- | --- | --- | --- | --- |
| **Dimensions of the full cochlea** | | | | | | |
| length* (mm) | 9.5 | 8.9** | 8.8** | 10.1 | 8.8 – 10.1 | 8.0 – 10.1^2,8,9^ |
| width* (mm) | 6.8 | 6.4** | 6.6** | 7.2 | 6.4 – 7.2 | 5.6 – 8.2^2,8–10^ |
| height (mm) | 4.2 | 3.7** | 3.6** | 4.0 | 3.6 – 4.2 | 3.3 – 4.8^2,8,10^ |
| number of turns | 2.8 | 2.6 | 2.7 | 2.6 | 2.6 – 2.8 | 2.2 – 2.9^2,8,10,11^ |
| volume* (mm^3^) | 81.3 | 73.6** | 75.8** | 102.7 | 73.6 – 102.7 | 50.8 – 118.5^11–14^ |
| **Dimensions of the intracochlear structures** | | | | | | |
|  | **Volume (mm3)** | | | | | |
| RWM | 0.49 | 0.63 | 0.52 | 0.37 | 0.37 – 0.63 |  |
| ST* | 30.9 | 29.1** | 29.6** | 44.2 | 29.1 – 44.2 | 23.0 – 50.0^9,15^ |
| SM* | 8. 9 | 8.6 | 8.8 | - | 8.6 – 8.9 |  |
| SV* | 25.1 | 21.1 | 23.3 | - | 21.1 – 25.1 |  |
| SV + SM* | 34.0 | 29.7 | 32.1 | 42.5 | 29.7 – 42.5 |  |
| CPB-OoC | 0.89 | 0.96 | 0.92 | 0.98 | 0.89 – 0.98 |  |
| OSL* | 2.4 | 2.0** | 2.0** | 2.7 | 2.0 – 2.7 |  |
| SL | 11.9 | 10.3 | 9.9 | 11.5 | 9.9 – 11.9 |  |
| SSL | 0.69 | 0.81** | 0.76** | 0.44 | 0.44 – 0.81 |  |
| RWA | 0.11 | 0.11 | 0.08 | 0.05 | 0.05 – 0.11 |  |
| RC | 1.4 | 1.5 | 1.7 | 1.7 | 1.4 – 1.7 |  |
|  | **Centerline length (mm)** | | | | | |
| ST | 31.8 | 31.7 | 31.9 | 36.0 | 31.7 – 36.0 | 28.4 – 30.7^15^ |
| SM | 44.3 | 43.8 | 48.2 | - | 43.8 – 48.2 | 35.4 – 43.6^16^ |
| SV* | 34.6 | 29.1** | 30.0** | - | 29.1 – 34.6 |  |
| CPB-OoC | 32.3 | 33.3 | 35.6 | 40.0 | 32.3 – 40.0 | 24.0 – 40.1^3,17–19^ |
| OSL* | 26.2 | 22.9** | 23.9** | 27.0 | 22.9 – 27.0 |  |
| SL | 54.8 | 54.5 | 57.8 | 62.1 | 54.5 – 62.1 |  |
| SSL | 29.4 | 35.3** | 34.4** | 41.5 | 29.4 – 41.5 |  |
| RWA | 4.2 | 5.5** | 5.8** | 3.0 | 3.0 – 5.8 |  |
| RC | 14.8 | 14.9 | 16.4 | 13.9 | 13.9 – 16.4 | 14.4 – 17.0^3,16–18,20^ |
|  | **Angular measurements (°)** | | | | | |
| RC - proximal end | 19 | 1.3** | 0.1** | 50 | 0.1 – 50 |  |
| RC - bulb | 697 | 709 | 663 | 696 | 663 – 709 | 684-704^16^ |
| SSL - distal end | 370 | 494** | 476** | 572 | 370 – 572 | 22^21^ |

**Supplementary Table 2. RWM thickness distribution in the four cochleae.** This table summarizes the measured values, graphically represented in ***Figure 3***. The columns on the left show the measured RWM thickness range with its respective mid-range value; the columns on the right represent the percentage of RWM area within each thickness range for each of the four cochleae.

| **thickness range (mm)** | **mid-range thickness (mm)** | **% RWM area in range** | | | |
| --- | --- | --- | --- | --- | --- |
|  |  | **IE_1L** | **IE_2L** | **IE_3R** | **IE_4R** |
| 0.019 - <0.057 | 0.038 | 3.2 | 0.7 | 1.9 | 1.1 |
| 0.057 - <0.095 | 0.076 | 24.4 | 14.4 | 37.5 | 25.7 |
| 0.095 - <0.132 | 0.113 | 29.1 | 37.2 | 28.2 | 29.2 |
| 0.132 - <0.170 | 0.151 | 15.7 | 29.0 | 15.9 | 27.6 |
| 0.170 - <0.208 | 0.189 | 16.4 | 13.3 | 11.7 | 14.5 |
| 0.208 - <0.246 | 0.227 | 11.2 | 5.4 | 3.3 | 1.8 |
| 0.246 - <0.284 | 0.265 | 0 | 0 | 1.6 | 0 |

**Supplementary Table 3. Overview of the sample characteristics.** All four samples, shown in ***Supplementary Figure 1***, were frozen one or two times before CECT imaging. The inner ear of *IE_2L* was snap-frozen in isopentane solution, cooled by liquid nitrogen to -160°C prior to imaging, while it was wrapped in two layers of parafilm foil. The other samples were frozen slowly at -20°C.

|  | **age at death (years)** | **gender** | **freezing cycles** | **freezing method, temp. (°C)** | **freezing time after death (h)** | **frozen storage duration (days)** | **frozen storage temp. (°C)** |
| --- | --- | --- | --- | --- | --- | --- | --- |
| IE_1L | 86 | female | 2 | slow, -20 | 18 | 106 | -20 |
| IE_2L | 72 | female | 1 | snap, -160 | 115 | 89 | -80 |
| IE_3R | 72 | female | 1 | slow, -20 | 115 | 152 | -20 |
| IE_4R | 71 | male | 2 | slow, -20 | 17 | 280 | -20 |

**Supplementary Table 4. Overview of the microCT acquisition parameters in the four samples.** The samples IE_2L and IE_3R were imaged with a long (LDD) and short (SDD) detector distance, whereby the short detector distance parameters were identical to IE_1L and IE_4R. The short detector distance datasets in the samples IE_2L and IE_3R were acquired only on day 3 and day 7 after submersion in Hf-WD POM ***(Supplementary Figure 2)***. After surgical opening of the samples IE_2L and IE_3R for histological processing, they were imaged in ‘fast scan’ (F) mode to assess for possible trauma ***(Supplementary Figure 3, CECT open cochlea)*** and to guide the position of the 2D histological sections (HIS), as visualized in ***Movie 1***. All other scans were carried out in ‘long scan’ (L) mode. Only for the scan with electrode in IE_4R, a filter (Pt-Au-coated Al 1.0 mm) was used.

| **sample** | **additional information** | **filter** | **voltage (keV)** | **current (µA)** | **voxel size (µm)** | **number of images** | **frame averaging** | **scan type** | **source-detector distance (mm)** | **exposure time (ms)** | **scanning time (hh:mm)** |
| --- | --- | --- | --- | --- | --- | --- | --- | --- | --- | --- | --- |
| IE_1L |  | - | 50 | 531 | 6.3 | 2400 | 3 | L | 266 | 500 | 01:26 |
| IE_2L | SDD | - | 50 | 531 | 6.3 | 2400 | 3 | L | 266 | 500 | 01:26 |
|  | LDD | - | 50 | 531 | 6.3 | 2400 | 3 | L | 475 | 750 | 02:06 |
|  | HIS | - | 50 | 531 | 6.3 | 2400 | - | F | 266 | 500 | 00:20 |
| IE_3R | SDD | - | 50 | 531 | 6.3 | 2400 | 3 | L | 266 | 500 | 01:26 |
|  | LDD | - | 50 | 531 | 6.3 | 2400 | 3 | L | 475 | 1000 | 02:46 |
|  | HIS | - | 50 | 531 | 6.3 | 2400 | - | F | 266 | 500 | 00:20 |
| IE_4R |  | - | 50 | 531 | 6.3 | 2400 | 3 | L | 266 | 500 | 01:26 |
| IE_4R  electr. |  | + | 100 | 265 | 6.3 | 2400 | 3 | L | 600 | 750 | 02:06 |

**Supplementary Table 5. Operator-dependency of the segmentation, based on the measured *RWM* volume in IE_3R.** The experienced operators, Op. 1 and Op. 2, had ≥ 2 years of experience with microCT image segmentation and a good knowledge of the 3D microanatomy of the RWM. The non-experienced operators, Op. 3 and Op. 4, had good general knowledge of the cochlear anatomy, but it was their first-time segmentation after receiving the training. For experienced operators, the inter-operator difference was comparable to the intra-operator difference, and was maximum 2% of the total RWM volume. The unexperienced operators had an equally small inter-operator difference with respect to each other, but both underestimated the volume of the RWM by 24%, compared to the experienced operators.

|  | **one experienced operator** | | **two experienced operators** | | **two non-experienced operators** | | **experienced**  **versus**  **non-experienced** | |
| --- | --- | --- | --- | --- | --- | --- | --- | --- |
| **measurements (mm^3^)** | Op. 1 | 0.523 | Op. 1 | 0.523 | Op. 3 | 0.407 | Op. 1 | 0.535 |
|  | Op. 1 | 0.535 | Op. 2 | 0.527 | Op. 4 | 0.415 | Op. 3 | 0.407 |
| **difference (mm^3^)** |  | 0.012 |  | 0.004 |  | 0.008 |  | 0.128 |
| **difference %** |  | 2 |  | 0.8 |  | 2 |  | 24 |

**MOVIES**

**
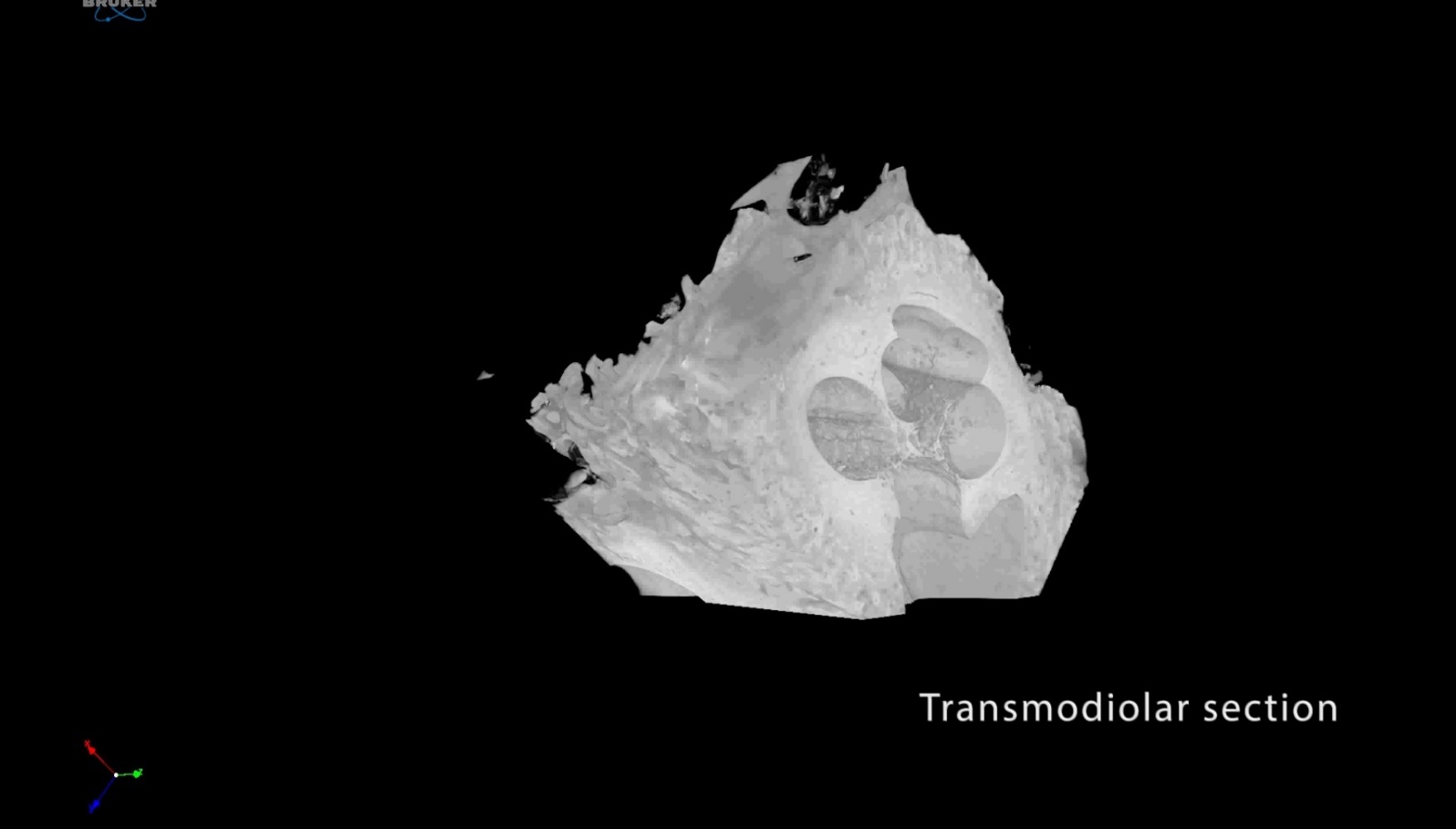
**

**Movie 1. Position of the transmodiolar and transbasal 2D histological cross-sections on the 3D CECT dataset in IE_2L.** *RWM: round window membrane; ST: scala tympani; SV: scala vestibuli.*

**
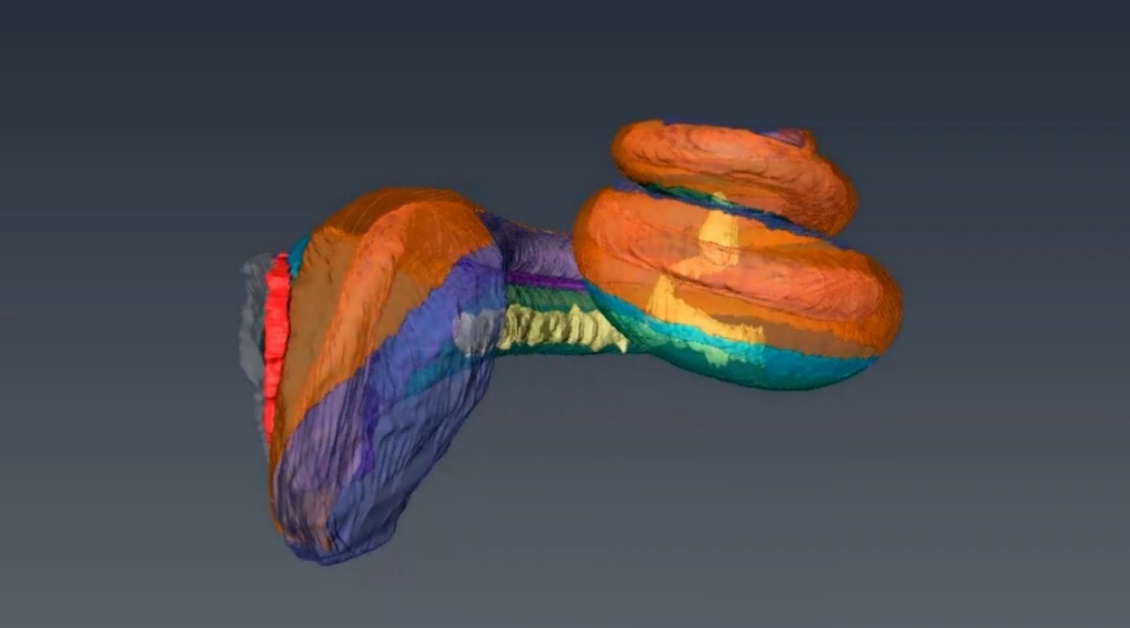
**

**Movie 2. 3D rendering of the intracochlear structures and compartments in IE_2L.** *CPB-OoC: cochlear partition bridge – organ of Corti complex; OSL: osseous spiral lamina; RC: Rosenthal’s canal; RWA: round window arch; RWM: round window membrane; SL: spiral ligament; SM: scala media; SSL: secondary spiral lamina; ST: scala tympani; SV: scala vestibuli.*


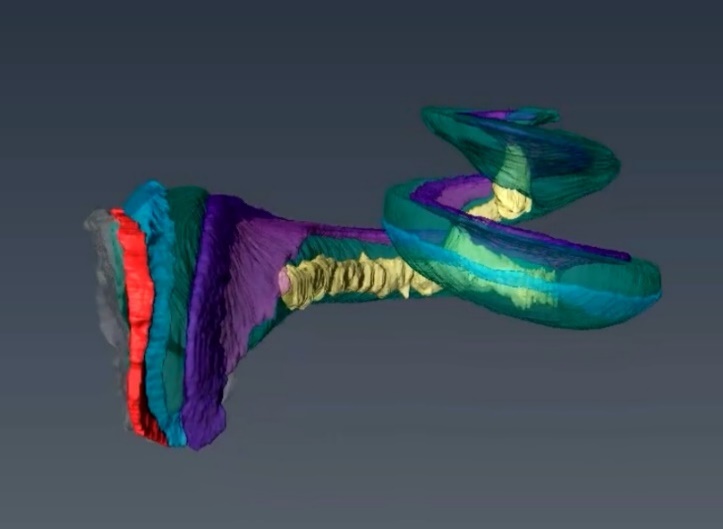


**Movie 3. The Rosenthal’s canal in IE_2L.** *CPB-OoC: cochlear partition bridge – organ of Corti complex; OSL: osseous spiral lamina; RC: Rosenthal’s canal; RWM: round window membrane; SL: spiral ligament; SM: scala media; ST: scala tympani; SV: scala vestibuli.*

**
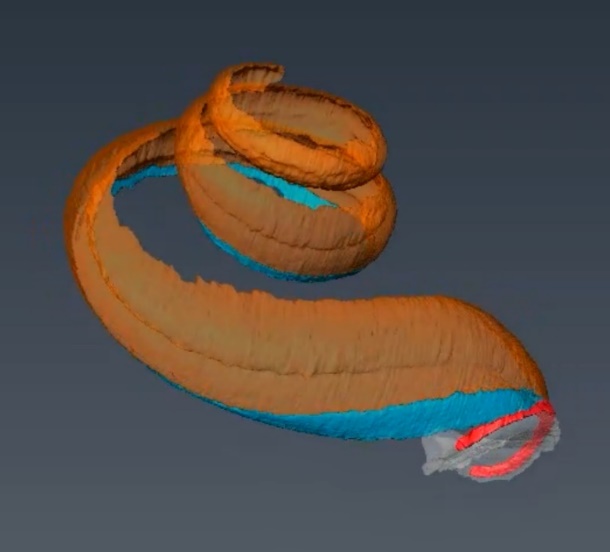
**

**Movie 4. The secondary spiral lamina and the round window arch in IE_2L.** *CPB-OoC: cochlear partition bridge – organ of Corti complex; OSL: osseous spiral lamina; RC: Rosenthal’s canal; RWA: round window arch; RWM: round window membrane; SL: spiral ligament; SM: scala media; SSL: secondary spiral lamina; ST: scala tympani; SV: scala vestibuli.*

**
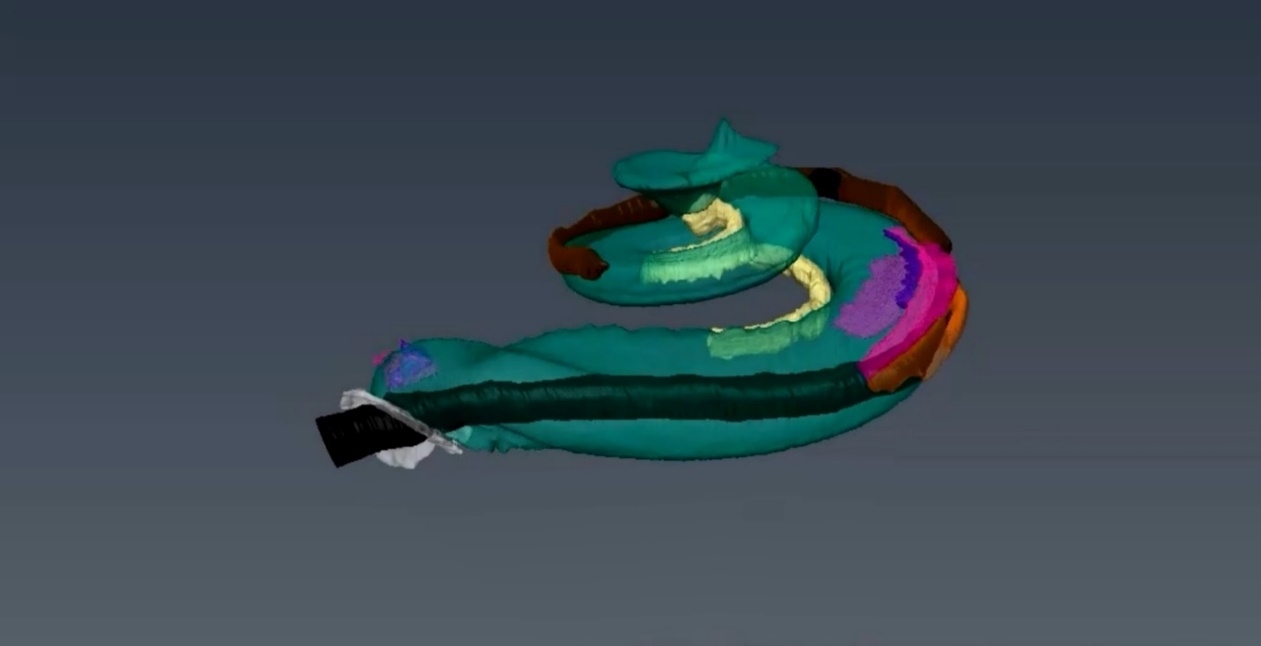
**

**Movie 5. Electrode insertion trauma in IE_4R.** The first two electrode insertions are hypothetical trajectory simulations, which could explain the mechanism of visualized trauma, and not based on CECT-proven electrode position. Note that the final insertion resulted in electrode transgression from the ST into the SL, causing trauma of the SL. *BM: basilar membrane; CPB-OoC: cochlear partition bridge – organ of Corti complex; OSL: osseous spiral lamina; RC: Rosenthal’s canal; RWM: round window membrane; SL: spiral ligament; ST: scala tympani.*

**References**

1. Agrawal, S. *et al.* The secondary spiral lamina and its relevance in cochlear implant surgery. *Ups. J. Med. Sci.* **123**, 9–18 (2018).

2. Shin, K.-J. *et al.* Quantitative Analysis of the Cochlea using Three-Dimensional Reconstruction based on Microcomputed Tomographic Images. *Anat. Rec.* **296**, 1083–1088 (2013).

3. Kawano, A., Seldon, H. L. & Clark, G. M. Computer-aided three-dimensional reconstruction in human cochlear maps: measurement of the lengths of organ of Corti, outer wall, inner wall, and Rosenthal’s canal. *Ann. Otol. Rhinol. Laryngol.* **105**, 701–9 (1996).

4. van den Boogert, T. *et al.* Optimization of 3D-Visualization of Micro-Anatomical Structures of the Human Inner Ear in Osmium Tetroxide Contrast Enhanced Micro-CT Scans. *Front. Neuroanat.* **12**, 41 (2018).

5. Brunschwig, A. S. & Salt, A. N. Fixation-induced shrinkage of Reissner’s membrane and its potential influence on the assessment of endolymph volume. *Hear. Res.* **114**, 62–68 (1997).

6. Edge, R. M. *et al.* Morphology of the unfixed cochlea. *Hear. Res.* **124**, 1–16 (1998).

7. Buytaert, J., Goyens, J., De Greef, D., Aerts, P. & Dirckx, J. Volume shrinkage of bone, brain and muscle tissue in sample preparation for micro-CT and light sheet fluorescence microscopy (LSFM). *Microsc. Microanal.* **20**, 1208–1217 (2014).

8. Avci, E., Nauwelaers, T., Lenarz, T., Hamacher, V. & Kral, A. Variations in microanatomy of the human cochlea. *J. Comp. Neurol.* **522**, 3245–61 (2014).

9. Dhanasingh, A., Swords, C., Bance, M., Van Rompaey, V. & Van de Heyning, P. Cochlear Size Assessment Predicts Scala Tympani Volume and Electrode Insertion Force- Implications in Robotic Assisted Cochlear Implant Surgery. *Front. Surg.* **8**, 723897 (2021).

10. Erixon, E., Högstorp, H., Wadin, K. & Rask-Andersen, H. Variational Anatomy of the Human Cochlea. *Otol. Neurotol.* **30**, 14–22 (2009).

11. Conde-Valverde, M. *et al.* The cochlea of the Sima de los Huesos hominins (Sierra de Atapuerca, Spain): New insights into cochlear evolution in the genus Homo. *J. Hum. Evol.* **136**, 102641 (2019).

12. Kendi, T. K., Arikan, O. K. & Koc, C. Volume of components of labyrinth: Magnetic resonance imaging study. *Otol. Neurotol.* **26**, 778–781 (2005).

13. Kirk, E. C. & Gosselin-Ildari, A. D. Cochlear Labyrinth Volume and Hearing Abilities in Primates. *Anat. Rec. Adv. Integr. Anat. Evol. Biol.* **292**, 765–776 (2009).

14. Takahashi, M. *et al.* Cochlear volume as a predictive factor for residual-hearing preservation after conventional cochlear implantation. *Acta Otolaryngol.* **138**, 345–350 (2018).

15. Al-Dhamari, I. *et al.* Automatic Estimation of Cochlear Duct Length and Volume Size. in *Bildverarbeitung für die Medizin* 55–55 (Springer, 2019). doi:10.1007/978-3-658-25326-4_15.

16. Danielian, A., Ishiyama, G., Lopez, I. A. & Ishiyama, A. Morphometric linear and angular measurements of the human cochlea in implant patients using 3-dimensional reconstruction. *Hear. Res.* **386**, 107874 (2020).

17. Sridhar, D., Stakhovskaya, O. & Leake, P. A. A Frequency-Position Function for the Human Cochlear Spiral Ganglion. *Audiol. Neurotol.* **11**, 16–20 (2006).

18. Li, H. *et al.* Three-dimensional tonotopic mapping of the human cochlea based on synchrotron radiation phase-contrast imaging. *Sci. Rep.* **11**, 1–8 (2021).

19. Helpard, L. *et al.* Three-Dimensional Modeling and Measurement of the Human Cochlear Hook Region: Considerations for Tonotopic Mapping. *Otol. Neurotol.* **42**, e658–e665 (2021).

20. Li, H. *et al.* Synchrotron Radiation-Based Reconstruction of the Human Spiral Ganglion: Implications for Cochlear Implantation. *Ear Hear.* **41**, 173–181 (2018).

21. Ekdale, E. G. Comparative Anatomy of the Bony Labyrinth (Inner Ear) of Placental Mammals. *PLoS One* **8**, e66624 (2013).
